# Human Papillomavirus Minor Capsid Protein L2 Mediates Intracellular Trafficking into and Passage beyond the Endoplasmic Reticulum

**DOI:** 10.1128/spectrum.01505-22

**Published:** 2022-05-24

**Authors:** Anthony V. Morante, Daniel Davidnan Baboolal, Xavier Simon, Elizabeth/Mark Cheng-Ying Pan, Patricio I. Meneses

**Affiliations:** a Department of Biological Sciences, Fordham University, Bronx, New York, USA; University of Manitoba

**Keywords:** HPV16, L2 protein, virus trafficking, endoplasmic reticulum, papillomavirus

## Abstract

Human papillomaviruses (HPVs) consist of two capsid proteins: major capsid protein L1 and minor capsid protein L2. The L2 protein has been shown to be involved in intracellular trafficking events that lead to the deposition of the viral DNA into the nucleus. In this study, we investigate the role of HPV16 L2 residues 43-DQILQ-47 during intracellular trafficking in human keratinocytes. We demonstrate that the highly conserved amino acids aspartic acid, isoleucine, and leucine are involved with the intracellular trafficking of the virus. Amino acid substitution of the isoleucine and leucine residues with alanine residues results in a significant decrease in infectivity of the pseudovirions without any changes to the binding or internalization of the virus. The pseudovirions containing these substitutions exhibit an altered trafficking pattern and do not deposit the viral pseudogenome into the nucleus. Instead, these mutated pseudovirions display a lack of interaction with syntaxin 18, an ER SNARE protein, are unable to progress past the endoplasmic reticulum (ER) and are redirected to the lysosomes. The results of this study help to elucidate the role and potential involvement of the 43-DQILQ-47 sequence during intracellular trafficking, specifically during trafficking beyond the ER.

**IMPORTANCE** High-risk types of human papillomaviruses (HPVs), such as HPV16, are highly associated with cervical, anogenital, and oropharyngeal cancers. The minor capsid protein L2 is essential for the intracellular trafficking of the viral DNA to the nucleus. This study investigates the role of amino acid residues 43-DQILQ-47 of the HPV16 L2 protein in the intracellular trafficking of the virus. Understanding how the virus traffics through the cell is a key factor in the development of additional preventative antiviral therapies. This study illustrates, through modification of the 43-DQILQ-47 sequence in pseudovirions, the importance of the 43-DQILQ-47 sequence in the trafficking of the virus beyond the endoplasmic reticulum.

## INTRODUCTION

Papillomaviruses are a family of nonenveloped, double-stranded DNA viruses that infect cutaneous and mucosal tissue (1–4). Low-risk types of human papillomavirus (HPV) are associated with warts, while high-risk types are linked to cervical, anogenital, and oropharyngeal cancers (3, 4). Specifically, HPV16, along with HPV18, is one of the most commonly found types involved with these associated cancers (5).

Upon entry, for HPV to deposit its viral DNA into the nucleus, it must traffic through the cell. The major capsid protein L1 is involved with the initial binding and internalization of the virus (6–9) and may also play a role in the intracellular trafficking (10, 11). The minor capsid protein L2 is found to be important in the trafficking of the viral particle toward the nucleus (12, 13). The L2 protein is also found to be involved in the packaging of the viral DNA (14). The L2 protein is necessary for infection as virus-like particles that contain only the L1 capsid protein carry significantly less viral DNA and are noninfectious (15–17).

It is important to understand the intracellular trafficking methods used by a virus during infection to identify potential antiviral targets. The trafficking pattern of HPV and the role L2 plays has made great progress in the last decade, although some areas, such as post-Golgi trafficking, remain elusive. To enter the cell, HPV is found to bind to heparan sulfate proteoglycans (HSPGs) (18–21), which initialize a conformational change that exposes the L2 N-terminus that is cleaved by furin (22). After internalization, HPV is found first in the early endosome (21, 23, 24), and later the late endosome (24–26), where the capsid undergoes a conformational change through the actions of cyclophilins (27, 28). It is possible that the unfolding of the virus is facilitated by the acidification process that occurs due to the low pH of the vesicles (25). Studies involving late endosomal/lysosomal enzyme inhibitors, such as NH_4_Cl or Bafilomycin A1, have associated the decreased enzymatic activity with decreased viral infection (29). It has been suggested that the L1 and L2 proteins would dissociate at the late endosome and the L2/DNA complex would progress toward the nucleus (28), although recent studies suggest that the L1 protein accompanies the L2 protein and the viral DNA throughout trafficking (10, 11, 30). Furin cleavage of L2 is found to be necessary for the movement of HPV from the late endosome (31, 32) to the Golgi apparatus via retrograde trafficking (24, 30, 32). Recent work from the DiMaio lab has implicated the role of γ-secretase in retrograde trafficking of HPV. It is observed that the C-terminus of L2 embeds itself into the endosome membrane to interact with a sorting complex known as retromer (33), which facilitates trafficking to the Golgi apparatus potentially through interaction with Rab7 GTPase and sorting nexin 17 (SNX17) (23). A recent study has elucidated the involvement of glutathione in the egression of viral particles from the Golgi apparatus (34). Although it has been demonstrated that HPV then travels to the endoplasmic reticulum (ER) (33, 35–37), the steps between association with the ER and entering the nucleus remain unclear. HPV infects upon nuclear breakdown during mitosis (38, 39). It has recently been determined that the HPV16 L2 protein forms a complex with Ran-binding protein 10, karyopherin alpha2, and the dynein light chain DYNLT3, which facilitates the nuclear import of the viral genome (40). The HPV16 L2 protein was also found to contain a central chromosome-binding region involved with binding to chromosomes during mitosis (41). After nuclear envelope reformation, the deposited DNA is observed to co-localize with the promyelocytic leukemia (PML) nuclear bodies during a successful infection (42, 43).

In previous studies, we had demonstrated the interaction between bovine papillomavirus type 1 (BPV1) L2 protein and ER-localized SNARE protein syntaxin 18 (35, 36), a protein involved in the vesicular sorting between the ER and Golgi apparatus (44). Residues 40-DKILK-44 of BPV1 L2 are important for the interaction with syntaxin 18. These L2 residues are expected to be located on the surface of the L2 protein and are exposed during infection, as shown by electron microscopy of antibody binding to the 40-DKILK-44 sequence (16, 35, 45). Antibody targeting of the 40-DKILK-44 residues neutralizes infection (35). Although the interaction with syntaxin 18 has been suggested, the role of these residues still remains unclear during HPV16 trafficking.

In this study, we focus on the homologous 43-DQILQ-47 sequence in the HPV16 L2 protein. Through substitution of the isoleucine and leucine residues with alanine via alanine scanning mutagenesis, we demonstrate that the mutant pseudovirions (PsVs) are noninfectious. We were interested in determining how the alteration in the L2 amino acid sequence would affect the trafficking of the PsVs. We show that the isoleucine-leucine mutant PsVs maintain a trafficking pattern similar to the wild-type L2 PsVs up until association with the ER. Instead of progressing into the nucleus, the mutant PsVs are instead redirected to the lysosome. The isoleucine-leucine mutant PsVs also exhibit a reduced interaction with syntaxin 18, which is observed in the wild-type PsVs.

## RESULTS

### ΔIL mutant PsVs are noninfectious and do not interfere with HPV16 binding or internalization in HaCaT cells.

**FIG 1:**
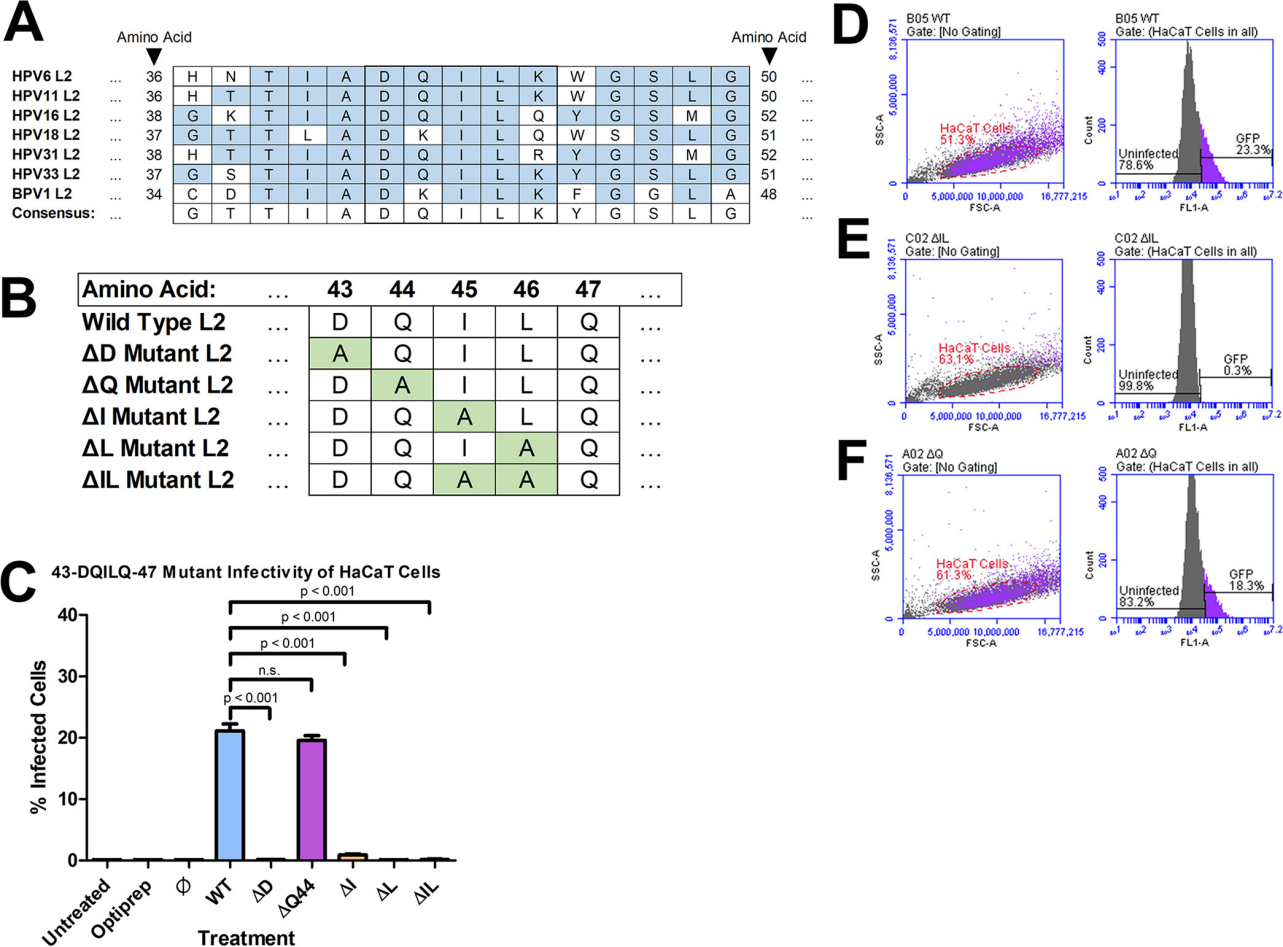
ΔIL mutant PsVs are noninfectious. (A) The 43-DQILQ-47 sequence is highly conserved across several types of HPV, including two low-risk (6 and 11) and four high-risk (16,18, 31, and 33). L2 protein sequences obtained from NCBI website (46). (B) Resulting amino acid sequences of PsVs after alanine scanning mutagenesis. ΔD mutant PsVs contain a substitution of L2 residue 43 with alanine. ΔQ mutant PsVs contain a substitution of L2 residue 44 with alanine. ΔI mutant PsVs contain a substitution of L2 residue 45 with alanine. ΔL mutant PsVs contain a substitution of L2 residue 46 with alanine. ΔIL mutant PsVs contain a substitution of L2 residues 45 and 46 with alanine. (C) Quantification of flow cytometry data for GFP expression. Pseudovirions were generated with plasmids where each individual amino acid in DQILQ sequence was substituted with an alanine by alanine scanning mutagenesis. Data is the average of three samples. Data is represented as mean ± SEM, *n* = 3. Individual statistical differences determined by Bonferroni’s post-test after significant ANOVA, α = 0.05, df = 8. (D–F) Flow cytometry data showing density plot with parent gate selecting HaCaT cells of interest (left) and histogram of cells within parent gate that express GFP (right) for (D) WT PsVs, (E) ΔIL mutant PsVs, and (F) ΔQ mutant PsVs.

We had previously shown that the BPV1 L2 residues 40-DKILK-44 were necessary for infection (35, 36). We focus on the homologous L2 residues 43-DQILQ-47 during HPV16 infection. The DQ/KILQ/K sequence is conserved across many types of HPV, including low-risk types HPV6 and HPV11, as well as high-risk types HPV16, HPV18, HPV31, and HPV33 (Fig. 1A). HPV L2 protein sequences were obtained from National Center for Biotechnology Information (NCBI) website (46). The isoleucine and leucine residues are highly conserved across nearly all HPV types. The only variation seen in the sequence is the presence of a glutamine or lysine flanking the isoleucine and leucine residues. For this study, we used alanine scanning mutagenesis to produce plasmids that encoded substituted forms of the HPV16 L2 protein. After PsV production, the resulting PsVs would contain an L2 protein with the aspartic acid in residue 43 substituted with an alanine residue (ΔD), the glutamine in residue 44 substituted with an alanine residue (ΔQ), the isoleucine in residue 45 substituted with an alanine residue (ΔI), the leucine in residue 46 substituted with an alanine residue (ΔL), or the isoleucine and leucine in residues 45 and 46 substituted with two alanine residues (ΔIL) (Fig. 1B).

The infectivity of the mutant PsVs in HaCaT cells was compared to PsVs carrying the wild-type (WT) L2 protein using flow cytometry (Fig. 1C to F). The percent infectivity for each PsV is the average of three independent samples. The ΔD, ΔI, and ΔL mutant PsVs had a significant average reduction in infectivity to 0.1%, 0.9%, and 0.1%, respectively (Fig. 1C; *P* < 0.001). The ΔQ mutant PsVs exhibited a similar average infectivity to the WT PsVs with 19.6% and 21.1%, respectively (Fig. 1C and D, and 1F). The ΔIL mutant PsVs had a significant loss in average infectivity to 0.17% (Fig. 1C and E; *P* < 0.001).

During our investigation, we noticed the isoleucine-leucine motif present at residues 45 and 46 in the 43-DQILQ-47 sequence. It has been suggested that isoleucine- and leucine-based motifs are involved in mediating the sorting of vesicles, particularly in the export from the Golgi apparatus as well as from the ER (47–50). ΔIL mutant PsVs had a comparable loss of infectivity to ΔI and ΔL mutant PsVs (Fig. 1C). We decided to focus our study on the ΔIL and ΔQ mutant PsVs.

**FIG 2:**
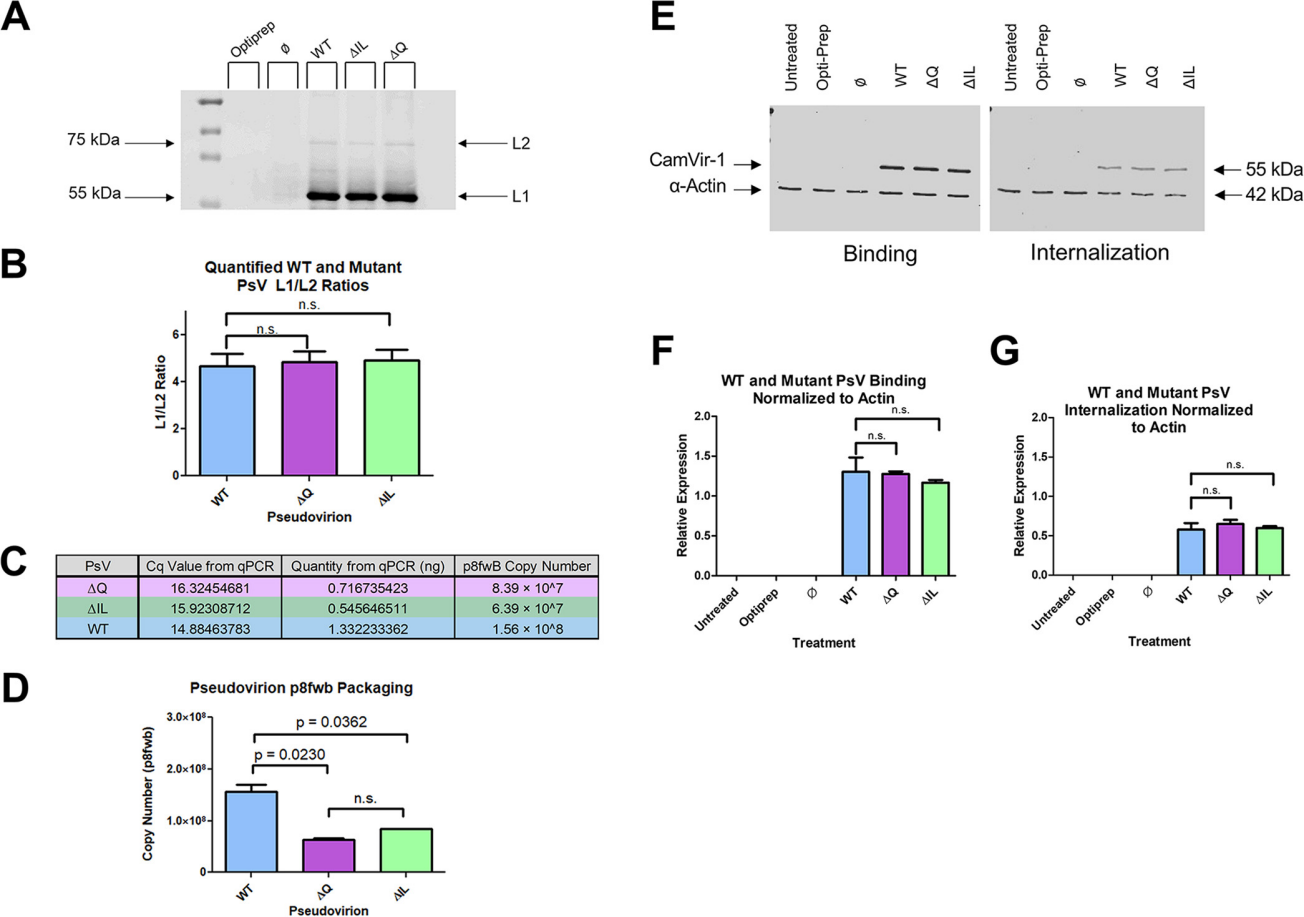
ΔIL mutation does not affect PsV formation, ability to package plasmid, binding, or internalization. (A) Western blot image of L1 and L2 proteins. Purified viral fractions were subjected to SDS-PAGE and incubated with L1 and L2 primary antibodies. Equal volumes of Optiprep and Ø plasmid preparation were loaded as controls. (B) L1 to L2 ratio based on Western blot image. Quantification of L1 and L2 bands was performed using ImageJ software. (C) qPCR results after targeting DNA extracted from viral fractions for p8fwB. Quantity of p8fwB was used to calculate p8fwB copy number in sample. (D) Copy number of p8fwB plasmid for WT, ΔIL mutant, and ΔQ mutant PsVs. Copy number is expressed as the amount of p8fwB plasmid (referred to as the pseudogenome) per μL of purified viral fraction, as determined by qPCR. Data is the average of two independent samples. Data is represented as mean ± SEM, *n* = 2. Individual statistical differences determined by Bonferroni’s post-test after significant ANOVA, α = 0.05, df = 2. (E) Western blot image of L1 and actin levels in HaCaT cells. Cell were harvested for lysates at 2 h after infection in lysis bffer to test binding ability of PsVs (LEFT). Cells were collected after trypsin treatment for 10 min before addition of lysis buffer to remove externally bound PsVs and test for internalized PsVs (RIGHT). (F) Normalization of L1 protein to actin protein for PsV binding. Normalization was performed using ImageJ software. (G) Normalization of L1 protein to actin protein for PsV internalization. Data is the average of three independent samples. Data is represented as mean ± SEM, *n* = 3. Individual statistical differences determined by Bonferroni’s post-test after significant ANOVA, α = 0.05, df = 2.

We assessed whether the ΔIL and ΔQ mutant PsVs contained the L1 and L2 proteins and were able to package the reporter plasmid. ΔIL and ΔQ mutant PsVs contained similar amounts of L1 and L2 proteins, comparable to the WT L2 PsVs, and each PsV had a similar L1/L2 ratio (Fig. 2A and B). ΔIL and ΔQ mutant PsVs were also able to package the reporter plasmid (Fig. 2C and D). ΔIL and ΔQ mutant PsVs exhibited a reduced pseudogenome copy number compared to the average copy number of 1.56 × 10^8^ viral genome equivalents (vge)/μL of purified viral fraction of WT L2 PsVs, with the ΔIL mutant PsVs containing an average of 6.39 × 10^7^ vge/μL of purified viral fraction and the ΔQ mutant PsVs containing an average of 8.39 × 10^7^ vge/μL of purified viral fraction (Fig. 2D; *P* = 0.0362 and *P* = 0.0230, respectively). ΔIL and ΔQ mutant PsVs had a comparable pseudogenome copy number. All future experiments were performed using an equal vge of 1500 for WT, ΔIL, and ΔQ mutant PsVs, which corresponds to approximately 15% GFP expression of WT PsVs via flow cytometry. We next assessed whether the loss of infection in the ΔIL mutant PsVs could be attributed to a loss of the ability of the PsVs to bind to or enter the cell. After 2 h of infection, the measure of bound PsVs was similar for WT, ΔIL mutant, and ΔQ mutant PsVs (Fig. 2E [Left] and F). L1 protein levels were normalized to actin (Fig. 2F and G). After 2 h of infection, trypsin was added to the HaCaT cells to remove externally bound PsVs. The measure of internalized L1 protein levels were similar for WT, ΔIL mutant, and ΔQ mutant PsVs (Fig. 2E [Right] and G). These data show that the loss of infection in the ΔIL mutant PsVs was not a result of a loss of binding or internalization.

### HPV16 PsVs associate with the early endosome 4 h postinfection in infected HaCaT cells.

**FIG 3:**
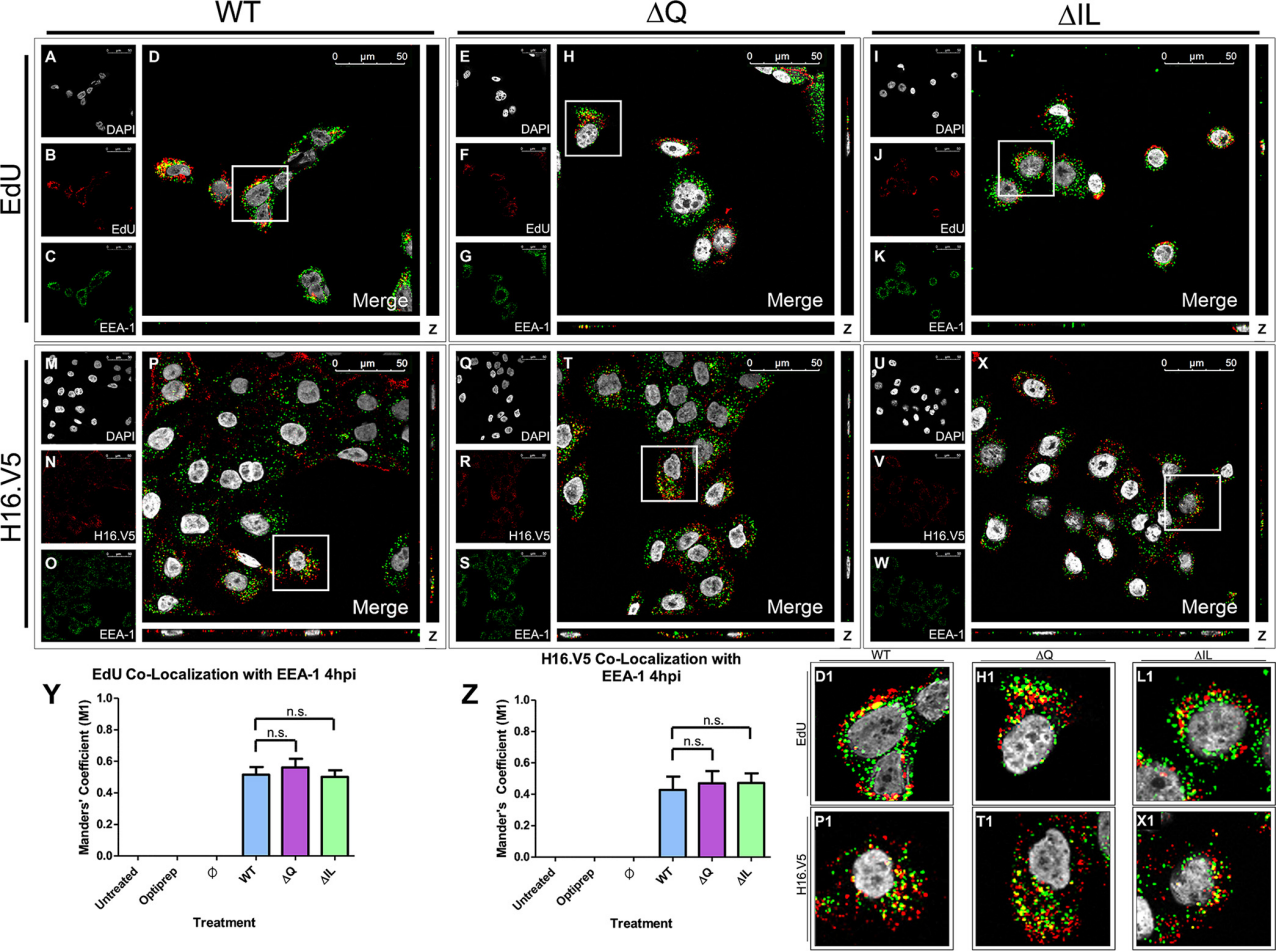
HPV16 pseudovirions associate with the early endosome 4 hpi. Cells were seeded onto coverslips and infected with WT, ΔQ mutant, or ΔIL mutant PsVs for 4 h before immunofluorescence slide preparation and antibody staining for either EdU and EEA-1 overlap (A–L) or H16.V5 and EEA-1 overlap (M–X) Representative immunofluorescence images are taken from the center of Z-stack image set. Top three panels represent EdU and EEA-1 signal for WT (A–D), ΔQ mutant (E–H), and ΔIL (I–L) mutant PsVs. (A, E, I) Nuclei stained with DAPI (gray). (B, F, J) Pseudogenome expressing EdU (red). (C, G, K) Early endosome stained with EEA-1 (green). (D, H, L) Merged image of all channels displaying colocalization of pseudogenome and EEA-1. (D1, H1, L1) Zoomed images of white square from merged channels. Bottom three panels represent H16.V5 and EEA-1 signal for WT (M–P), ΔQ mutant (Q–T), and ΔIL mutant PsVs (U–X). (M, Q, U) Nuclei stained with DAPI (gray). (N, R, V) H16.V5 representing HPV16 L1 protein (red). (O, S, W) Early endosome stained with EEA-1 (green). (P, T, X) Merged image of all channels displaying colocalization of H16.V5 and EEA-1. (P1, T1, X1) Zoomed images of white square from merged channels. (Y-Z) M1 coefficient for (Y) pseudogenome (EdU) or (Z) H16.V5 overlapping early endosome (EEA-1) at 4 h postinfection for untreated, Optiprep, Ø plasmid preparation, WT PsVs, ΔIL mutant PsVs, and ΔQ mutant PsVs. Data is the average of six independent confocal scans for each condition, each with the Manders’ M1 coefficient of four slices averaged together from the center of the Z-stack. Data is represented as mean ± SEM, *n* = 6. M1 coefficient was determined using the JACoP plugin for ImageJ. Individual statistical differences determined by Bonferroni’s post-test after significant ANOVA, α = 0.05, df = 5.

In order to determine whether the loss of infectivity of the ΔIL mutant PsVs were a result of a loss of association of the PsVs with the early endosome, HaCaT cells were infected with HPV16 PsVs containing an EdU-labeled pseudogenome. It has been shown that HPV16 PsVs will be in the early endosome up until 4 h postinfection (hpi) in HaCaT cells (21, 23, 24). DAPI was used for visualization of the nuclei (gray), the Click-It reaction for the EdU-labeled DNA (red), and an anti-EEA-1 antibody for the early endosome (green) (Fig. 3A to L). Trafficking of EdU-labeled PsVs containing WT L2 protein (Fig. 3A to D), ΔQ mutant (Fig. 3E to H), and ΔIL mutant (Fig. 3I to L) was visualized using immunofluorescence microscopy. Colocalization of the pseudogenome and EEA-1 was observed 4 hpi for the WT, ΔQ mutant, and ΔIL mutant PsVs (Fig. 3D, D1, H, H1, L, and L1). The average percentage of EdU-labeled pseudogenomes overlapping with EEA-1 was approximately 55% for the WT, ΔQ mutant, and ΔIL mutant PsVs, as indicated by the M1 coefficient (Fig. 3Y).

**FIG 4:**
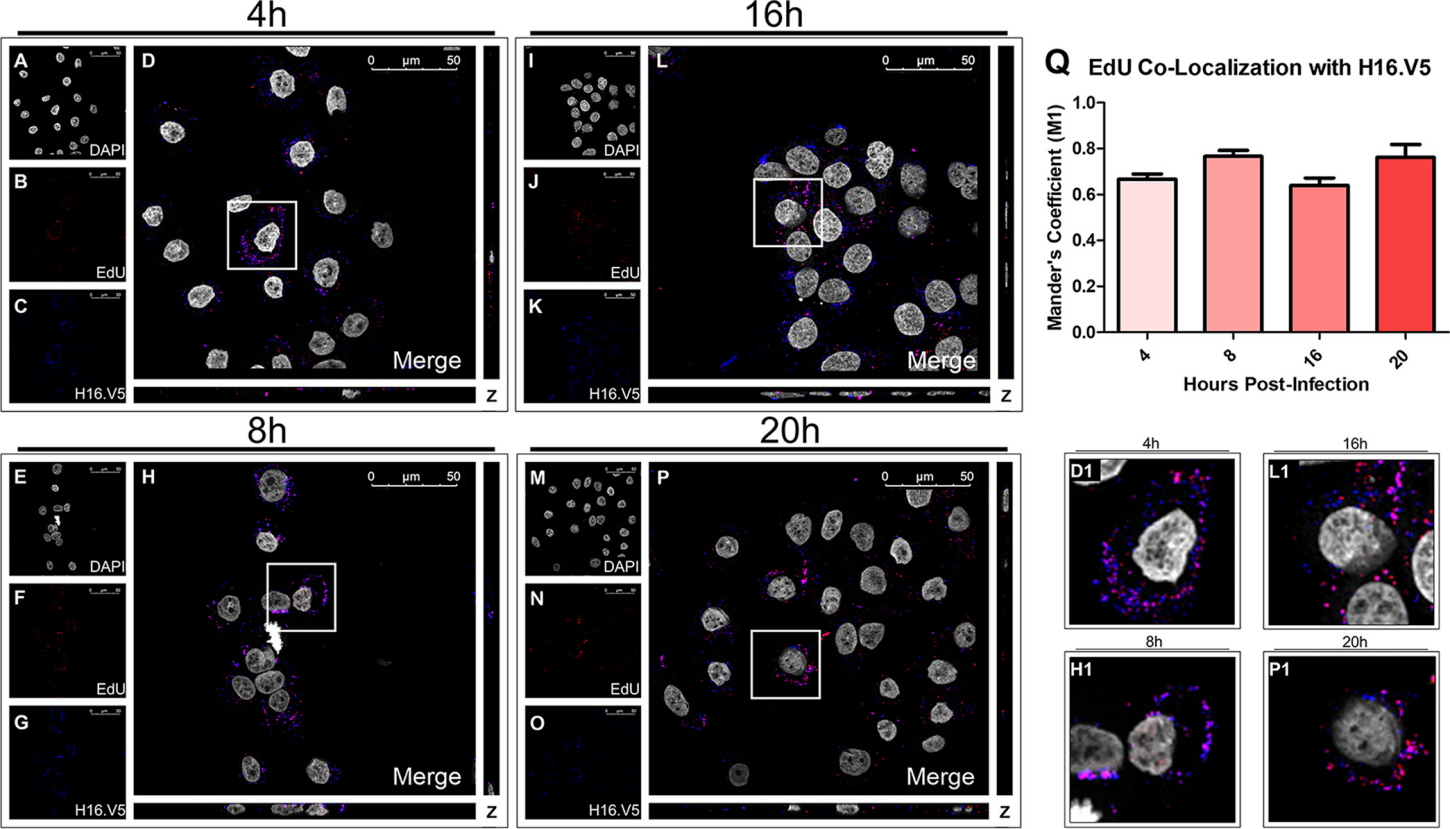
EdU signal corresponds to HPV viral particles. Cells were seeded onto coverslips and infected with WT PsVs for 4 h (A–D), 8 h (E–H), 16 h (I–L), or 20 h (M–P) before immunofluorescence slide preparation and antibody staining. Representative immunofluorescence images are taken from the center of Z-stack image set. (A, E, I, M) Nuclei stained with DAPI (gray). (B, F, J, N) Pseudogenome expressing EdU (red). (C, G, K, O) L1 protein stained with H16.V5 (green). (D, H, L, P) Merged image of all channels displaying colocalization of pseudogenome and L1. (D1, H1, L1, P1) Zoomed images of white square from merged channels. (Q) M1 coefficient for pseudogenome (EdU) overlapping L1 protein (H16.V5) at 4, 8, 16, and 20 h postinfection for untreated, Optiprep, Ø plasmid preparation, WT PsVs. Data is the average of six independent confocal scans for each condition, each with the Manders’ M1 coefficient of four slices averaged together from the center of the Z-stack. Data is represented as mean ± SEM, *n* = 6. M1 coefficient was determined using the JACoP plugin for ImageJ. Individual statistical differences determined by Bonferroni’s post-test after significant ANOVA, α = 0.05, df = 5.

To verify that the EdU signal corresponded to EdU-labeling of PsV pseudogenomes, we stained with H16.V5, an anti-L1 antibody, and observed colocalization between the EdU and H16.V5 signals for the WT PsVs (Fig. 4A to D and D1). Colocalization between EdU and H16.V5 was found to be approximately 65%, indicated by the M1 coefficient (Fig. 4Q). Colocalization was determined using the percentage of overlap of EdU to H16.V5. To avoid a loss of H16.V5 signal from the denaturing of the capsid proteins, as noted by Day et al. (32), we incubated with H16.V5 primary antibody before Click-It reaction treatment.

The accompaniment of the L1 protein with the L2/vDNA complex post-egression from the late endosome has been suggested recently (10, 11, 30). Since a similar colocalization between EdU and EEA-1 for the WT and mutant PsVs as well as between EdU and H16.V5 was observed, we sought to determine whether the capsid protein L1 attached to the WT and mutant PsVs would follow a similar organelle trafficking pattern to that of the pseudogenome. DAPI was used for visualization of the nuclei (gray), H16.V5 for the HPV16 L1 protein (red), and an anti-EEA-1 antibody for the early endosome (green) (Fig. 3M to X). Trafficking of the L1 capsid protein attached to PsVs containing WT L2 protein (Fig. 3M to P), ΔQ mutant (Fig. 3Q to T), and ΔIL mutant (Fig. 3U to X) was visualized using immunofluorescence microscopy. Colocalization of H16.V5 and EEA-1 was observed 4 hpi for the WT, ΔQ mutant, and ΔIL mutant PsVs (Fig. 3P, P1, T, T1, X, and X1). The average percentage of H16.V5 overlapping with EEA-1 was approximately 50% for the WT, ΔQ mutant, and ΔIL mutant PsVs, as indicated by the M1 coefficient (Fig. 3Z).

### HPV16 PsVs associate with the late endosome 8 h postinfection in infected HaCaT cells.

**FIG 5:**
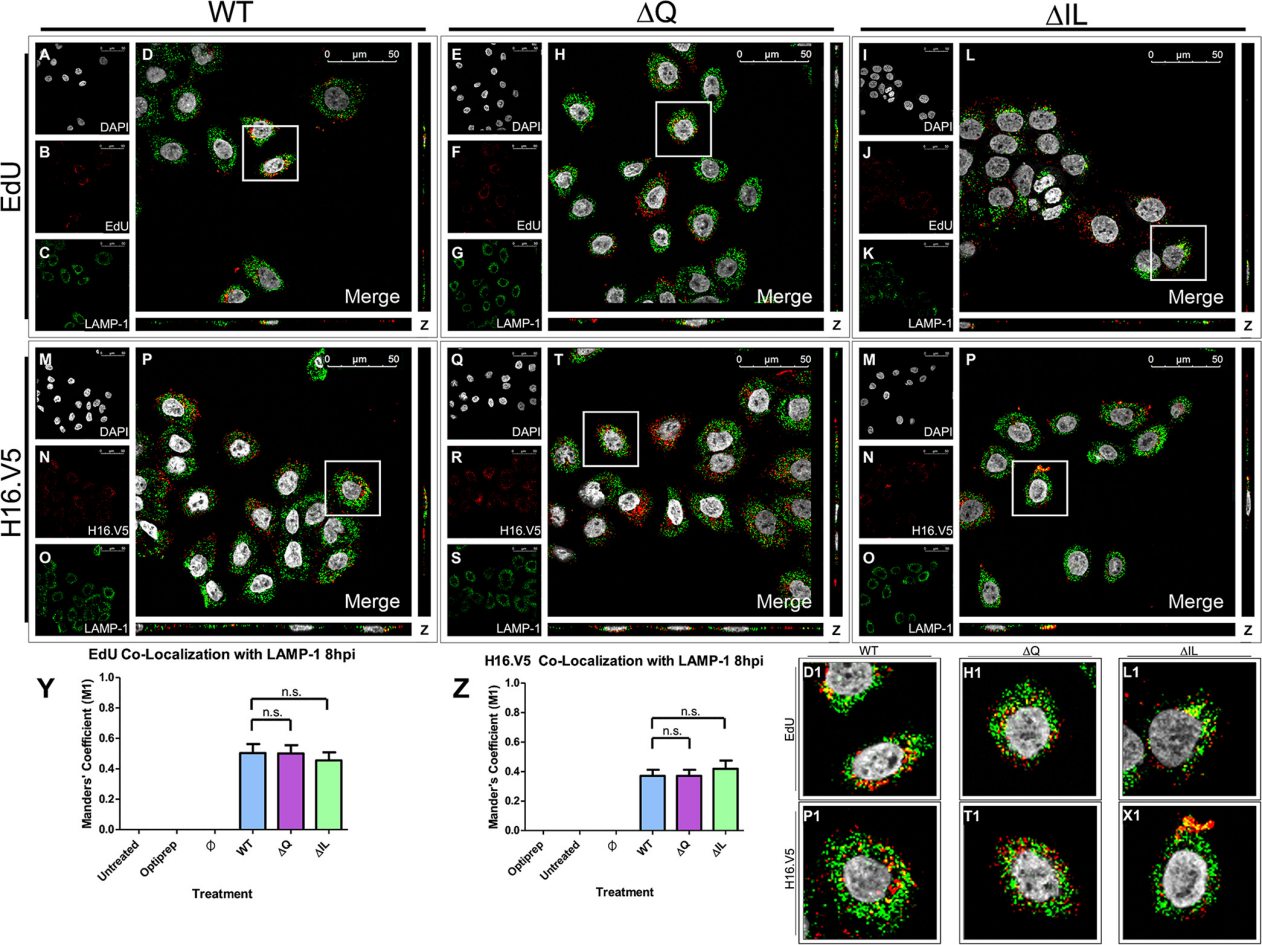
HPV16 pseudovirions associate with the late endosome 8 hpi. Cells were seeded onto coverslips and infected with WT, ΔQ mutant, or ΔIL mutant PsVs for 8 h before immunofluorescence slide preparation and antibody staining for either EdU and LAMP-1 overlap (A–L) or H16.V5 and LAMP-1 overlap (M–X) Representative immunofluorescence images are taken from the center of Z-stack image set. Top three panels represent EdU and LAMP-1 signal for WT (A-D), ΔQ mutant (E–H), and ΔIL mutant (I–L) PsVs. (A, E, I) Nuclei stained with DAPI (gray). (B, F, J) Pseudogenome expressing EdU (red). (C, G, K) Late endosome stained with LAMP-1 (green). (D, H, L) Merged image of all channels displaying colocalization of pseudogenome and LAMP-1. (D1, H1, L1) Zoomed images of white square from merged channels. Bottom three panels represent H16.V5 and LAMP-1 signal for WT (M-P), ΔQ mutant (Q–T), and ΔIL mutant (U–X) PsVs. (M, Q, U) Nuclei stained with DAPI (gray). (N, R, V) H16.V5 representing HPV16 L1 protein (red). (O, S, W) Late endosome stained with LAMP-1 (green). (P, T, X) Merged image of all channels displaying colocalization of H16.V5 and LAMP-1. (P1, T1, X1) Zoomed images of white square from merged channels. (Y-Z) M1 coefficient for (Y) pseudogenome (EdU) or (Z) H16.V5 overlapping late endosome (LAMP-1) at 8 h postinfection for untreated, Optiprep, Ø plasmid preparation, WT PsVs, ΔQ mutant PsVs, and ΔIL mutant PsVs. Data is the average of six independent confocal scans for each condition, each with the Manders’ M1 coefficient of four slices averaged together from the center of the Z-stack. Data is represented as mean ± SEM, *n* = 6. M1 coefficient was determined using the JACoP plugin for ImageJ. Individual statistical differences determined by Bonferroni’s post-test after significant ANOVA, α = 0.05, df = 5.

As the colocalization of the pseudogenome and early endosome were comparable at 4 hpi, we next sought to determine the next step in the trafficking of the pseudogenome. HPV16 PsVs are found to begin co-localizing with the late endosomal/lysosomal marker LAMP-1 around 8 hpi in HaCaT cells (24, 25) and HeLa cells (26). HaCaT cells were infected with the HPV16 PsVs that contained an EdU-labeled pseudogenome. DAPI was used for visualization of the nuclei (gray), the Click-It reaction for the EdU-labeled DNA (red), and an anti-LAMP-1 antibody for the late endosome/lysosome (green) (Fig. 5A to L). Trafficking of EdU-labeled PsVs containing WT L2 protein (Fig. 5A to D), ΔQ mutant (Fig. 5E to H), and ΔIL mutant (Fig. 5I to L) was visualized using immunofluorescence microscopy. Colocalization of the EdU-labeled pseudogenome and LAMP-1 was observed at 8 hpi for the WT, ΔQ mutant, and ΔIL mutant PsVs (Fig. 5D, D1, H, H1, L, and L1). The average percentage of overlap of the EdU-labeled pseudogenomes with LAMP-1 was approximately 50% for the WT, ΔQ mutant, and ΔIL mutant PsVs, as indicated by the M1 coefficient (Fig. 5Y). We observed colocalization between EdU and H16.V5 8 hpi of approximately 75%, as indicated by the M1 coefficient (Fig. 4E–H, H1, and Q).

Colocalization of H16.V5 and the early endosome was comparable for the WT and mutant PsVs at 4 hpi. We next observed the colocalization of H16.V5 with the late endosome/lysosomal marker LAMP-1 in HaCaT cells 8 hpi. DAPI was used for visualization of the nuclei (gray), H16.V5 for the HPV16 L1 protein (red), and an anti-LAMP-1 antibody for the late endosome/lysosome (green) (Fig. 5M to X). Trafficking of the L1 capsid protein attached to PsVs containing WT L2 protein (Fig. 5M to P), ΔQ mutant (Fig. 5Q to T), and ΔIL mutant (Fig. 5U to X) was visualized using immunofluorescence microscopy. Colocalization of H16.V5 and LAMP-1 was observed 8 hpi for the WT, ΔQ mutant, and ΔIL mutant PsVs (Fig. 5P, P1, T, T1, X, and X1). The average percentage of H16.V5 overlapping with LAMP-1 was approximately 40% for the WT, ΔQ mutant, and ΔIL mutant PsVs, as indicated by the M1 coefficient (Fig. 5Z).

### HPV16 PsVs associate with the Golgi apparatus 16 h postinfection in infected HaCaT cells.

**FIG 6:**
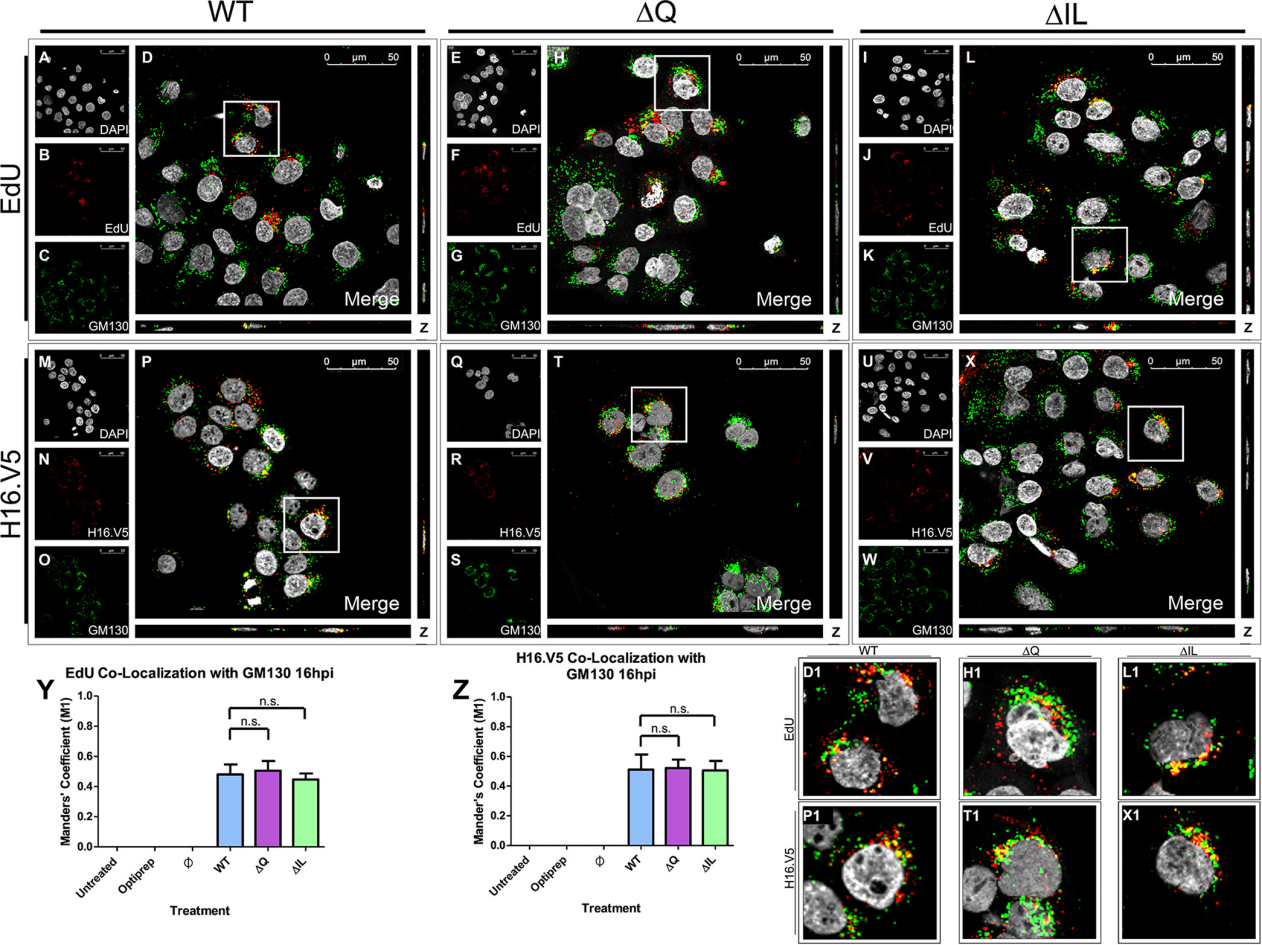
HPV16 pseudovirions associate with the Golgi apparatus 16 hpi. Cells were seeded onto coverslips and infected with WT, ΔQ mutant, or ΔIL mutant PsVs for 16 h before immunofluorescence slide preparation and antibody staining for either EdU and GM130 overlap (A–L) or H16.V5 and GM130 overlap (M–X) Representative immunofluorescence images are taken from the center of Z-stack image set. Top three panels represent EdU and GM130 signal for WT (A–D), ΔQ mutant (E–H), and ΔIL mutant (I–L) PsVs. (A, E, I) Nuclei stained with DAPI (gray). (B, F, J) Pseudogenome expressing EdU (red). (C, G, K) *cis-*Golgi stained with GM130 (green). (D, H, L) Merged image of all channels displaying colocalization of pseudogenome and GM130. (D1, H1, L1) Zoomed images of white square from merged channels. Bottom three panels represent H16.V5 and GM130 signal for WT (M-P), ΔQ mutant (Q-T), and ΔIL mutant (U-X) PsVs. (M, Q, U) Nuclei stained with DAPI (gray). (N, R, V) H16.V5 representing HPV16 L1 protein (red). (O, S, W) *cis*-Golgi stained with GM130 (green). (P, T, X) Merged image of all channels displaying colocalization of H16.V5 and GM130. (P1, T1, X1) Zoomed images of white square from merged channels. (Y-Z) M1 coefficient for (Y) pseudogenome (EdU) or (Z) H16.V5 overlapping *cis*-Golgi (GM130) at 16 h postinfection for untreated, Optiprep, Ø plasmid preparation, WT PsVs, ΔQ mutant PsVs, and ΔIL mutant PsVs. Data is the average of six independent confocal scans for each condition, each with the Manders’ M1 coefficient of four slices averaged together from the center of the Z-stack. Data is represented as mean ± SEM, *n* = 6. M1 coefficient was determined using the JACoP plugin for ImageJ. Individual statistical differences determined by Bonferroni’s post-test after significant ANOVA, α = 0.05, df = 5.

The trafficking pattern of the ΔIL and ΔQ mutants continued to be comparable to that of the WT at 8 hpi. In order to observe the trafficking of the pseudogenome post-8 h, HaCaT cells were infected with the HPV16 PsVs that contained an EdU-labeled pseudogenome. HPV16 PsVs have been found to co-localize with the Golgi apparatus between 8 hpi and 18 hpi in HaCaT cells (24, 30, 32). DAPI was used for visualization of the nuclei (gray), the Click-It reaction for the EdU-labeled DNA (red), and an anti-GM130 antibody for the *cis*-Golgi (green) (Fig. 6A to L). Trafficking of EdU-labeled PsVs containing WT L2 protein (Fig. 6A to D), ΔQ mutant (Fig. 6E to H), and ΔIL mutant (Fig. 6I to L) was visualized using immunofluorescence microscopy. Colocalization of the EdU-labeled pseudogenome and GM130 was observed at 16 hpi for the WT, ΔQ mutant, and ΔIL mutant PsVs (Fig. 6D, D1, H, H1, L, and L1). The average percentage of overlap of the EdU-labeled pseudogenomes with GM130 was approximately 50% for the WT, ΔQ mutant, and ΔIL mutant PsVs, as indicated by the M1 coefficient (Fig. 6Y). Colocalization between EdU and H16.V5 16 hpi was observed to be approximately 60%, as indicated by the M1 coefficient (Fig. 4I–L, L1, and Q).

H16.V5 and the late endosome co-localized at similar levels for the WT and mutant PsVs at 8 hpi. To continue to the next step in the trafficking pathway, we observed the colocalization of H16.V5 with the *cis*-Golgi marker GM130 at 16 hpi in HaCaT cells. DAPI was used for visualization of the nuclei (gray), H16.V5 for the HPV16 L1 protein (red), and an anti-GM130 antibody for the *cis*-Golgi (green) (Fig. 6M to X). Trafficking of the L1 capsid protein attached to PsVs containing WT L2 protein (Fig. 6M to P), ΔQ mutant (Fig. 6Q to T), and ΔIL mutant (Fig. 6U to X) was visualized using immunofluorescence microscopy. Colocalization of H16.V5 and GM130 was observed 16 hpi for the WT, ΔQ mutant, and ΔIL mutant PsVs (Fig. 6P, P1, T, T1, X, and X1). The average percentage of H16.V5 overlapping with GM130 was approximately 50% for the WT, ΔQ mutant, and ΔIL mutant PsVs, as indicated by the M1 coefficient (Fig. 6Z).

### HPV16 PsVs associate with the endoplasmic reticulum membrane 20 h postinfection in infected HaCaT cells.

**FIG 7:**
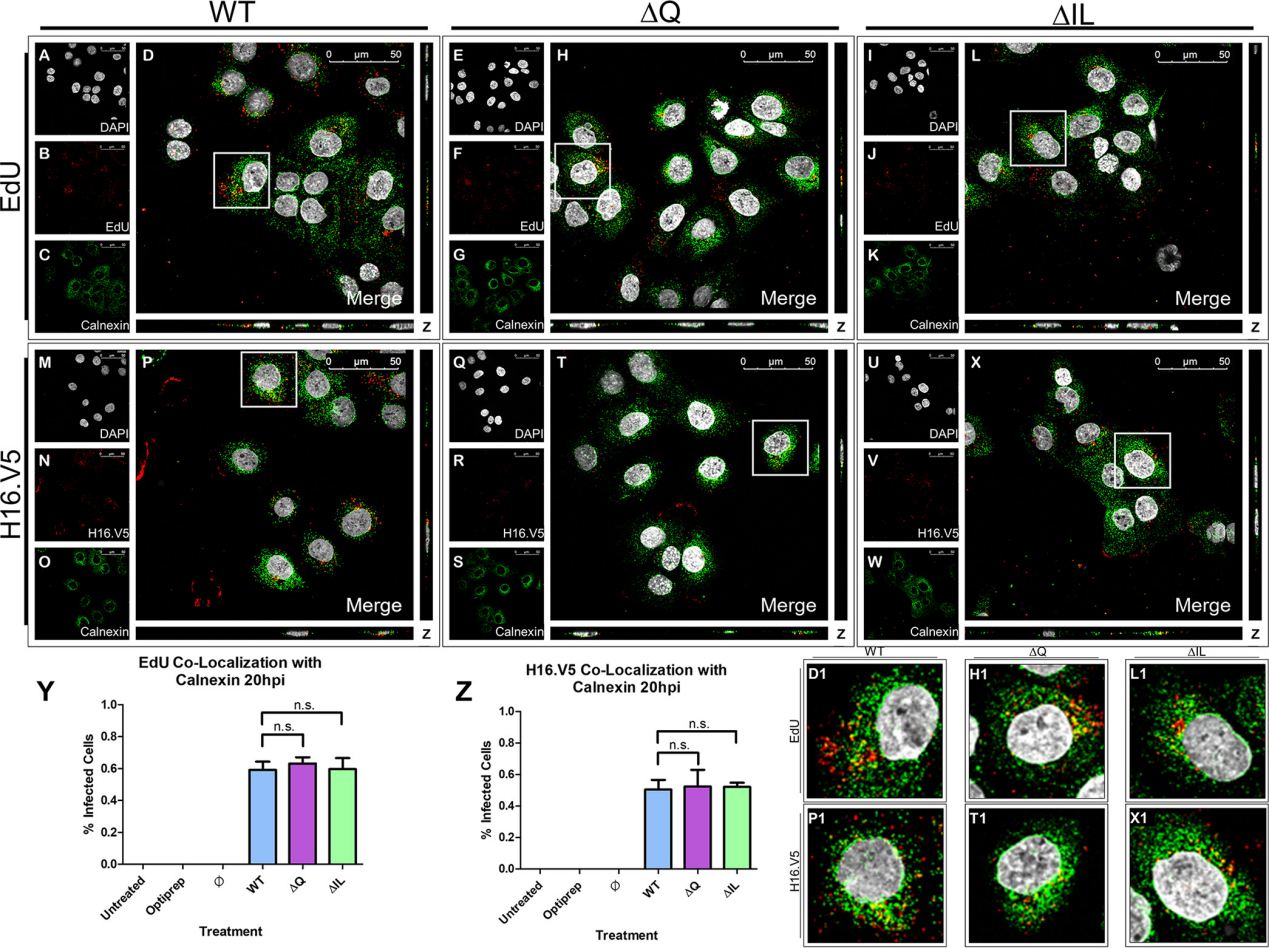
HPV16 pseudovirions associate with the endoplasmic reticulum 20 hpi. Cells were seeded onto coverslips and infected with WT, ΔQ mutant, or ΔIL mutant PsVs for 20 h before immunofluorescence slide preparation and antibody staining for either EdU and calnexin overlap (A–L) or H16.V5 and calnexin overlap (M-X) Representative immunofluorescence images are taken from the center of Z-stack image set. Top three panels represent EdU and calnexin signal for WT (A–D), ΔQ mutant (E-H), and ΔIL mutant (I-L) PsVs. (A, E, I) Nuclei stained with DAPI (gray). (B, F, J) Pseudogenome expressing EdU (red). (C, G, K) ER stained with calnexin (green). (D, H, L) Merged image of all channels displaying colocalization of pseudogenome and calnexin. (D1, H1, L1) Zoomed images of white square from merged channels. Bottom three panels represent H16.V5 and calnexin signal for WT (M-P), ΔQ mutant (Q-T), and ΔIL mutant (U-X) PsVs. (M, Q, U) Nuclei stained with DAPI (gray). (N, R, V) H16.V5 representing HPV16 L1 protein (red). (O, S, W) ER stained with calnexin (green). (P, T, X) Merged image of all channels displaying colocalization of H16.V5 and calnexin. (P1, T1, X1) Zoomed images of white square from merged channels. (Y-Z) M1 coefficient for (Y) pseudogenome (EdU) or (Z) H16.V5 overlapping ER membrane (calnexin) at 20 h postinfection for untreated, Optiprep, Ø plasmid preparation, WT PsVs, ΔQ mutant PsVs, and ΔIL mutant PsVs. Data is the average of six independent confocal scans for each condition, each with the Manders’ M1 coefficient of four slices averaged together from the center of the Z-stack. Data is represented as mean ± SEM, *n* = 6. M1 coefficient was determined using the JACoP plugin for ImageJ. Individual statistical differences determined by Bonferroni’s post-test after significant ANOVA, α = 0.05, df = 5.

After observing comparable colocalization of the EdU-labeled pseudogenome and the Golgi apparatus 16 hpi, we sought to observe the trafficking of the pseudogenome at the next step of infection. HaCaT cells were infected with the HPV16 PsVs that contained an EdU-labeled pseudogenome. It has been shown that HPV16 PsVs will traffic to the ER around 20 hpi in COS-7 cells (35, 36), HeLa cells (33), and HaCaT cells (37). We first assessed whether the WT and mutant PsVs would reach the ER membrane. We observed the colocalization of the WT and mutant PsVs with the transmembrane ER chaperone protein calnexin. DAPI was used for visualization of the nuclei (gray), the Click-It reaction for the EdU-labeled DNA (red), and an anti-calnexin antibody for the endoplasmic reticulum (green) (Fig. 7A to L). Trafficking of EdU-labeled PsVs containing WT L2 protein (Fig. 7A to D), ΔQ mutant (Fig. 7E to H), and ΔIL mutant (Fig. 7I to L) was visualized using immunofluorescence microscopy. Colocalization of the EdU-labeled pseudogenome and calnexin was observed at 20 hpi for the WT, ΔQ mutant, and ΔIL mutant PsVs (Fig. 7D, D1, H, H1, L, and L1). The average percentage of overlap of the EdU-labeled pseudogenomes with calnexin was approximately 60% for the WT, ΔQ mutant, and ΔIL mutant PsVs, as indicated by the M1 coefficient (Fig. 7Y).

Colocalization between EdU and H16.V5 16 hpi was observed to be approximately 75%, as indicated by the M1 coefficient (Fig. 4M–P, P1, and Q). As H16.V5 and the Golgi-apparatus had similar levels of colocalization for the WT and mutant PsVs, we next observed the colocalization of H16.V5 with calnexin at 20 hpi in HaCaT cells. DAPI was used for visualization of the nuclei (gray), H16.V5 for the HPV16 L1 protein (red), and an anti-calnexin antibody for the ER (green) (Fig. 7M to X). Trafficking of the L1 capsid protein attached to PsVs containing WT L2 protein (Fig. 7M to P), ΔQ mutant (Fig. 7Q to T), and ΔIL mutant (Fig. 7U to X) was visualized using immunofluorescence microscopy. Colocalization of H16.V5 and GM130 was observed 16 hpi for the WT, ΔQ mutant, and ΔIL mutant PsVs (Fig. 7P, P1, T, T1, X, and X1). The average percentage of H16.V5 overlapping with calnexin was approximately 50% for the WT, ΔIL mutant, and ΔQ mutant PsVs, as indicated by the M1 coefficient (Fig. 7Z). Our data supports the findings that during HPV16 PsV trafficking, PsVs will traffic in proximity to the ER (33, 35–37).

### ΔIL mutant PsVs have a decreased association with luminal ER protein GRP78 20 h postinfection in HaCaT cells.

**FIG 8:**
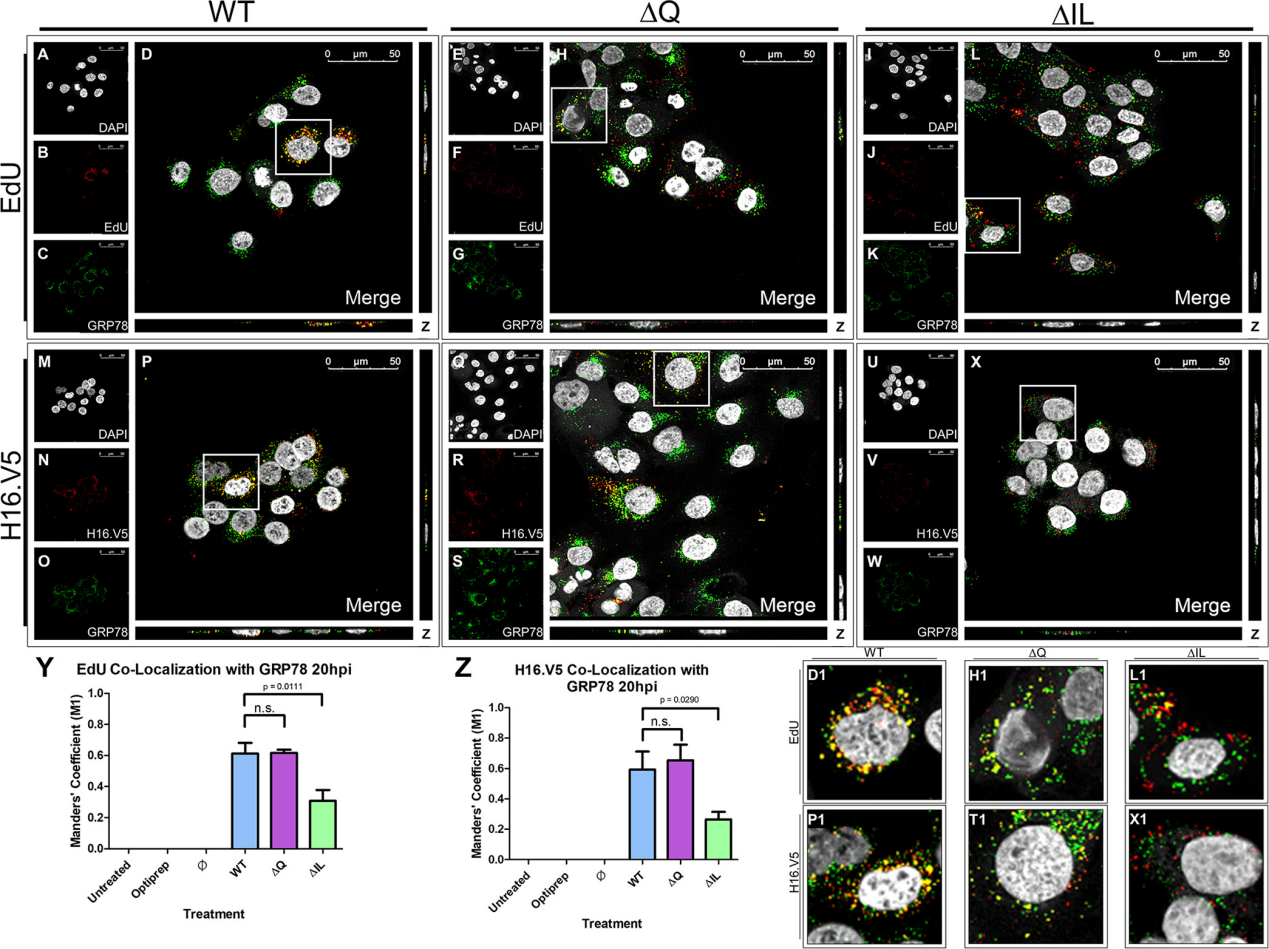
ΔIL mutant PsVs have a decreased association with luminal ER protein GRP78 20 h postinfection. Cells were seeded onto coverslips and infected with WT, ΔQ mutant, or ΔIL mutant PsVs for 20 h before immunofluorescence slide preparation and antibody staining for either EdU and GRP78 overlap (A-L) or H16.V5 and GRP78 overlap (M-X) Representative immunofluorescence images are taken from the center of Z-stack image set. Top three panels represent EdU and GRP78 signal for WT (A-D), ΔQ mutant (E-H), and ΔIL mutant (I-L) PsVs. (A, E, I) Nuclei stained with DAPI (gray). (B, F, J) Pseudogenome expressing EdU (red). (C, G, K) ER stained with GRP78 (green). (D, H, L) Merged image of all channels displaying colocalization of pseudogenome and GRP78. (D1, H1, L1) Zoomed images of white square from merged channels. Bottom three panels represent H16.V5 and GRP78 signal for WT (M–P), ΔQ mutant (Q–T), and ΔIL mutant (U–X) PsVs. (M, Q, U) Nuclei stained with DAPI (gray). (N, R, V) H16.V5 representing HPV16 L1 protein (red). (O, S, W) ER stained with GRP78 (green). (P, T, X) Merged image of all channels displaying colocalization of H16.V5 and GRP78. (P1, T1, X1) Zoomed images of white square from merged channels. (Y-Z) M1 coefficient for (Y) pseudogenome (EdU) or (Z) H16.V5 overlapping ER (GRP78) at 20 h postinfection for untreated, Optiprep, Ø plasmid preparation, WT PsVs, ΔQ mutant PsVs, and ΔIL mutant PsVs. Data is the average of six independent confocal scans for each condition, each with the Manders’ M1 coefficient of four slices averaged together from the center of the Z-stack. Data is represented as mean ± SEM, *n* = 6. M1 coefficient was determined using the JACoP plugin for ImageJ. Individual statistical differences determined by Bonferroni’s post-test after significant ANOVA, α = 0.05, df = 5.

As the WT and mutant PsVs exhibited a similar association with the ER membrane, we next sought to determine whether the mutation in the ΔIL mutant PsVs would affect the ability of the PsVs to enter the ER and associate with a luminal ER protein. GRP78, also called BiP, is a luminal ER chaperone protein in the heat shock protein-70 (HSP70) family that HPV PsVs are found to associate with (33, 51). DAPI was used for visualization of the nuclei (gray), the Click-It reaction for the EdU-labeled DNA (red), and an anti-GRP78 antibody for the endoplasmic reticulum (green) (Fig. 8A to L). Trafficking of EdU-labeled PsVs containing WT L2 protein (Fig. 8A to D), ΔQ mutant (Fig. 8E to H), and ΔIL mutant (Fig. 8I to L) was visualized using immunofluorescence microscopy. Colocalization of the EdU-labeled pseudogenome and GRP78 was observed at 20 hpi for the WT and ΔQ mutant (Fig. 8D, D1, H, and H1). The average percentage of overlap of the EdU-labeled pseudogenomes with GRP78 was approximately 60% for the WT and ΔQ mutant PsVs, as indicated by the M1 coefficient (Fig. 8Y). The ΔIL mutant PsVs exhibited a significant decrease in colocalization with GRP78 with approximately 30% (Fig. 8L, L1, and Y; *P* = 0.0111).

A similar result was observed in the trafficking pattern of the L1 protein and GRP78. DAPI was used for visualization of the nuclei (gray), H16.V5 for the HPV16 L1 protein (red), and an anti-GRP78 antibody for the ER (green) (Fig. 8M to X). Trafficking of the L1 capsid protein attached to PsVs containing WT L2 protein (Fig. 8M to P), ΔQ mutant (Fig. 8Q to T), and ΔIL mutant (Fig. 8U to X) was visualized using immunofluorescence microscopy. Colocalization of H16.V5 and GRP78 was observed 20 hpi for the WT and ΔQ mutant (Fig. 8P, P1, T, and T1). The average percentage of H16.V5 overlapping with GRP78 was approximately 60% for the WT and ΔQ mutant PsVs, as indicated by the M1 coefficient (Fig. 8Z). Similarly to the colocalization between the pseudogenome of the ΔIL mutant PsVs and GRP78, the colocalization between the L1 capsid protein of the ΔIL mutant PsVs and GRP78 was significantly reduced to approximately 25% (Fig. 8X, X1, and Z; *P* = 0.0290). The data for the pseudogenome colocalization with GRP78 and the L1 capsid protein colocalization with GRP78 together support the idea that there is an alteration in the trafficking pattern for the ΔIL mutant PsVs beyond the ER membrane as ΔIL mutant PsVs show a reduced association with the luminal ER protein GRP78.

### ΔIL mutant PsVs do not overlap with the PML bodies 48 h postinfection, but instead overlap with the lysosome.

As the trafficking pattern for the WT and ΔQ mutant PsVs remained consistent at each of the observed time points while the ΔIL mutant PsVs displayed an altered trafficking pattern, we next tested whether the PsVs would co-localize with the PML nuclear bodies in the nucleus. HPV will deposit the viral DNA into the nucleus during nuclear envelope break down (38, 39). As found by Day et al., multiple viruses, including papillomaviruses, are found to associate with PML nuclear bodies once the nuclear envelope reforms (42). EdU-labeled PsVs and PML nuclear body colocalization is present in HeLa cells at 40 hpi and in HaCaT cells as early as 1 hpi (32, 52).

**FIG 9:**
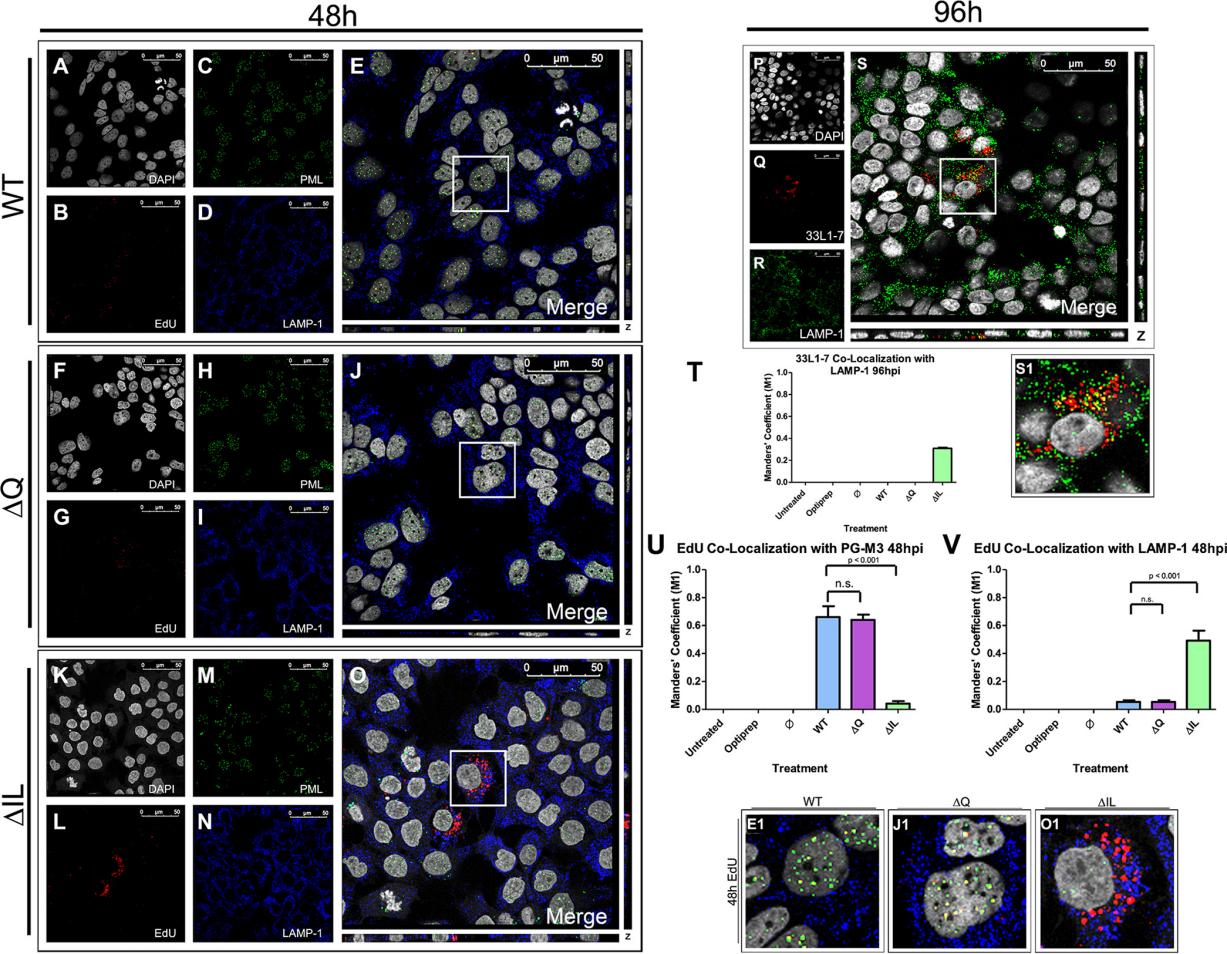
ΔIL mutant PsVs do not enter the nucleus 48 hpi and are instead deferred to the lysosomes. Cells were seeded onto coverslips and infected with WT (A–E), ΔIL mutant (F–J), or ΔQ mutant (K–O) PsVs for 48 h (A–O) or 96h (P–S) before immunofluorescence slide preparation and antibody staining. Representative immunofluorescence images are taken from the center of Z-stack image set. (A, F, K) Nuclei stained with DAPI (gray). (B, G, L) Pseudogenome expressing EdU (red). (C, H, M) Lysosomes stained with LAMP-1 (blue) (D, I, N) PML bodies stained with PG-M3 (green). (E, J, O) Merged image of all channels displaying EdU, LAMP-1, and PG-M3. (E1, J1, O1) Zoomed images of white square from merged channels. (P) Nuclei staining with DAPI (gray). (Q) 33L1-7 representing unfolded L1 protein (red). (R) Lysosomes stained with LAMP-1 (blue). (S) Merged image of all channels displaying colocalization of 33L1-7 and lysosomes. (S1) Zoomed images of white square from merged channel. (T-V) M1 coefficient for (T) 33L1-7 overlapping lysosomes (LAMP-1) or pseudogenome (EdU) overlapping (U) PML bodies (PG-M3) or (V) lysosomes (LAMP-1) at 48 h postinfection for untreated, Optiprep, Ø plasmid preparation, WT PsVs, ΔIL mutant PsVs, and ΔQ mutant PsVs. Data is the average of six independent confocal scans for each condition, each with the Manders’ M1 coefficient of four slices averaged together from the center of the Z-stack. Data is represented as mean ± SEM, *n* = 6. M1 coefficient was determined using the JACoP plugin for ImageJ. Individual statistical differences determined by Bonferroni’s post-test after significant ANOVA, α = 0.05, df = 5.

A recent study had found that treatment of HeLa and HaCaT cells with human defensin 5 reduced viral trafficking to the Golgi apparatus and instead redirected the viral particles to the lysosome (53). We next wanted to determine whether the altered viral trafficking through the ER exhibited by the ΔIL mutant PsVs also resulted in a redirection of the viral particles to the lysosome. HaCaT cells were infected with the HPV16 PsVs that contained an EdU-labeled pseudogenome. DAPI was used for visualization of the nuclei (gray), the Click-It reaction for the EdU-labeled DNA (red), an anti-LAMP-1 antibody for the lysosome (blue), and a PG-M3 antibody for the PML bodies (green) (Fig. 9A to O). Trafficking of EdU-labeled PsVs containing WT L2 protein (Fig. 9A to E), ΔQ mutant (Fig. 9F to J), and ΔIL mutant (Fig. 9K to O) was visualized using immunofluorescence microscopy. Colocalization of the EdU-labeled pseudogenome and the PML bodies was observed for the WT and ΔQ mutant PsVs at approximately 60% (Fig. 9E, E1, J, J1, and U). Colocalization of the EdU-labeled pseudogenome and the PML bodies was significantly reduced to less than 5% for the ΔIL mutant PsVs, which did not appear to co-localize with the PML nuclear bodies (Fig. 9O, O1, and U). Instead, colocalization was observed between the EdU-labeled pseudogenome and LAMP-1 for the ΔIL mutant PsVs with approximately 50% overlap (Fig. 9V). We observed very little EdU signal outside of the nucleus for WT and ΔQ mutant PsVs 48 hpi while the ΔIL mutant PsVs appeared to aggregate outside of the nucleus.

To determine the fate of the ΔIL mutant PsVs once they are redirected to the lysosome, we performed immunofluorescent microscopy using the anti-L1 antibody 33L1-7, which recognizes an unexposed epitope, as a marker for capsid disassembly (54). HaCaT cells were infected with ΔIL mutant HPV16 PsVs for 96 h. DAPI was used for visualization of the nuclei (gray), 33L1-7 for L1 (red), and an anti-LAMP-1 antibody for the lysosome (green) (Fig. 9P to S). After 96 hpi, 33L1-7 is found to co-localize with the lysosome approximately 30% (Fig. 9S, S1, and T), according to the M1 coefficient. This suggests that the redirected ΔIL mutant PsVs are sent to the lysosome for degradation. WT and ΔQ mutant PsVs did not exhibit any presence of 33L1-7 at 96 hpi (data not shown).

The data in this study show that lack of colocalization with the PML nuclear bodies by the ΔIL mutant PsVs is correlated with a loss of infection. The ΔQ mutant PsVs exhibited a comparable infectivity to the WT L2 PsVs as well as a comparable amount of colocalization with the PML nuclear bodies. While the WT and ΔQ mutant PsVs are able to progress beyond the ER and co-localize with the PML nuclear bodies, the ΔIL mutant PsVs are observed to remain outside of the nucleus and co-localize with the lysosome. These data suggest that the progression of HPV16 PsVs beyond the ER, and ultimately the deposition of the viral DNA into the nucleus, is dependent upon the 43-DQILQ-47 residues, particularly, isoleucine and leucine, of the L2 protein.

### WT HPV16 PsVs interact with syntaxin 18 20 h postinfection and the interaction is lost in the ΔIL mutant PsVs.

After we observed a reduction in the association between both the pseudogenome and GRP78 and the L1 capsid protein and GRP78 for the ΔIL mutant PsVs, we were interested in determining whether syntaxin 18 was involved in the trafficking of HPV16 PsVs. Syntaxin 18 is a SNARE protein involved in the fusion of vesicles into the ER membrane (44). In a previous study using BPV1, we found that antibody targeting to the 40-DKILK-44 region of BPV1 L2 interferes with the interaction between L2 and syntaxin 18 (35).

**FIG 10:**
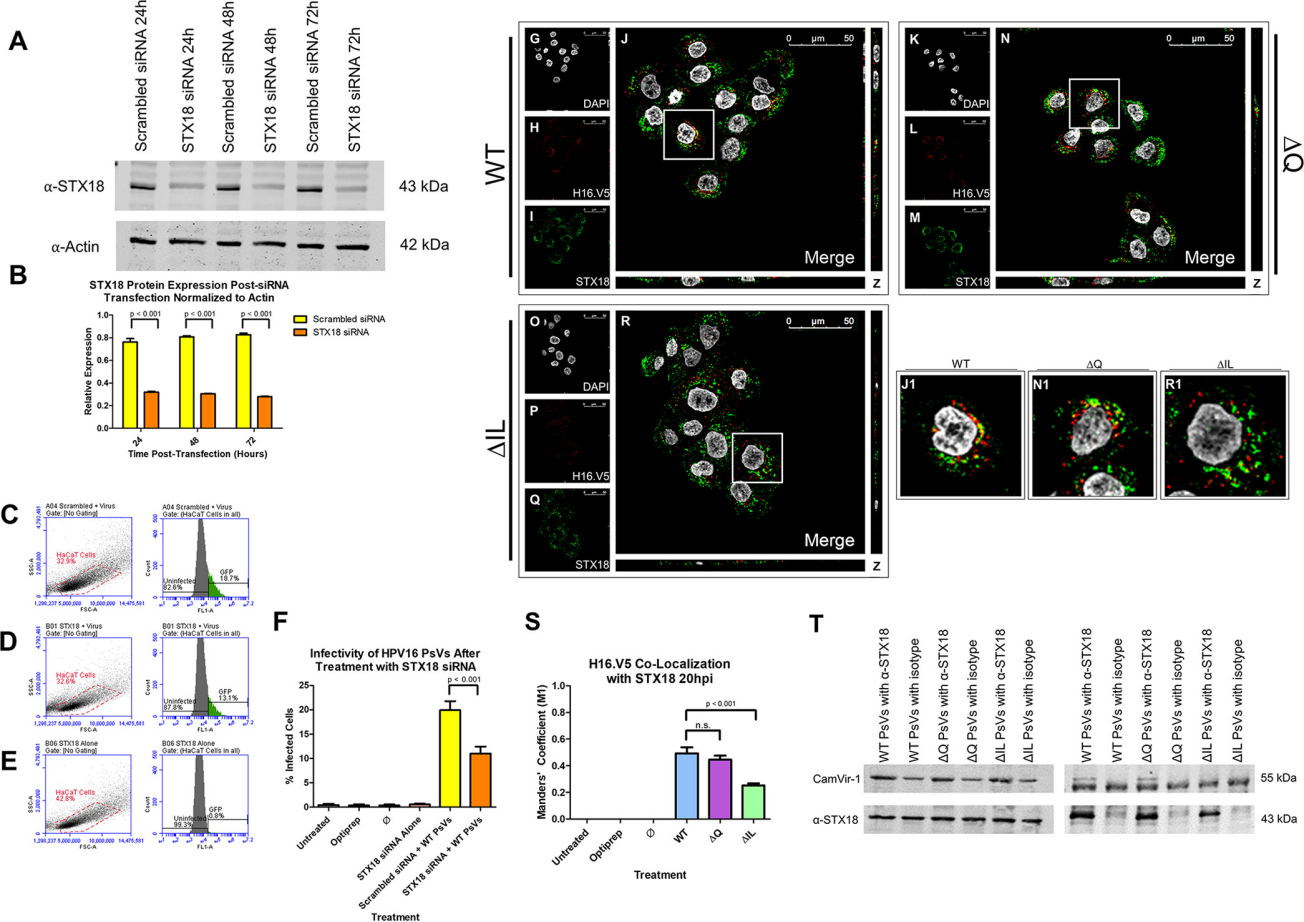
WT HPV16 PsVs interact with syntaxin 18 20 h postinfection and the interaction is lost in the ΔIL mutant PsVs. (A) Western blot image of syntaxin 18 siRNA 24, 48, and 72 h posttransfection for actin (lower) and syntaxin 18 (upper). HaCaT cells were transfected with either syntaxin 18 siRNA or scrambled siRNA control. (B) Normalization of syntaxin 18 protein levels to actin. Data is the average of three independent samples. Data is represented as mean ± SEM, *n* = 3. (C–E) Flow cytometry data showing density plot with parent gate selecting HaCaT cells of interest (left) and histogram of cells within parent gate that express GFP (right) for (C) cells transfected with scrambled siRNA and infected with WT PsVs, (D) cells transfected with STX18 siRNA and infected with WT PsVs and (E) cells transfected with STX18 siRNA without infection. (F) Quantification of flow cytometry data for GFP expression. Data is the average of three independent experiments. Data is represented as mean ± SEM, *n* = 3. (G–R) Cells were seeded onto coverslips and infected with WT (A-D), ΔQ mutant (E-H), or ΔIL mutant (I–L) PsVs for 20 h before immunofluorescence slide preparation and antibody staining for H16.V5 and syntaxin 18 overlap. Representative immunofluorescence images are taken from the center of Z-stack image set. (G, K, O) Nuclei stained with DAPI (gray). (H, L, P) H16.V5 representing L1 (red). (I, M, Q) syntaxin 18 (green). (J, N, R) Merged image of all channels displaying colocalization of H16.V5 and syntaxin 18. (J1, N1, R1) Zoomed images of white square from merged channels. (S) M1 coefficient for H16.V5 overlapping syntaxin 18 at 20 h postinfection for untreated, Optiprep, Ø plasmid preparation, WT PsVs, ΔQ mutant PsVs, and ΔIL mutant PsVs. Data is the average of six independent confocal scans for each condition, each with the Manders’ M1 coefficient of four slices averaged together from the center of the Z-stack. Data is represented as mean ± SEM, *n* = 6. M1 coefficient was determined using the JACoP plugin for ImageJ. Individual statistical differences determined by Bonferroni’s post-test after significant ANOVA, α = 0.05, df = 5. (T) Western blot image of co-immunoprecipitation for syntaxin 18 and L1 protein (CamVir-1). HaCaT cells were infected with WT, Q mutant, or IL mutant PsVs for 20 h before harvesting in lysis buffer. Samples were incubated with either anti-syntaxin 18 antibody or rabbit IgG isotype. Image displays flow through for samples (LEFT) or eluates (RIGHT).

As syntaxin 18 has yet to be implicated in HPV16 trafficking, we were interested in whether siRNA knockdown of syntaxin 18 will affect the infectivity of HPV16 PsVs. To confirm knockdown of syntaxin 18 in HaCaT cells, we tested syntaxin 18 protein levels 24, 48, and 72 h after initial siRNA transfection in HaCaT cells (Fig. 10A). Syntaxin 18 protein expression was normalized to actin (Fig. 10B). Knockdown of syntaxin 18 was consistent up to 72 h after initial transfection with a syntaxin-18-to-actin ratio of approximately 0.30, which was significantly reduced compared to the syntaxin-18-to-actin ratio of approximately 0.80 observed in the scrambled-siRNA treated cells (Fig. 10A and B). Once knockdown of syntaxin 18 was confirmed, we infected HaCaT cells with HPV16 WT PsVs 24 h after initial siRNA transfection. Infectivity of cells treated with scrambled siRNA was observed to be approximately 20%, on average, which was significantly reduced in cells treated with syntaxin 18 siRNA at approximately 11%, on average (Fig. 10C to F; *P* < 0.001). No infectivity was observed in cells treated with syntaxin 18 siRNA alone (Fig. 10E).

As siRNA knockdown of syntaxin 18 appeared to decrease infectivity of HPV16 PsVs, we next performed immunofluorescent microscopy to assess whether the WT and mutant PsVs associate with syntaxin 18 in HaCaT cells at 20 hpi. DAPI was used for visualization of the nuclei (gray), H16.V5 for the L1 protein (red), and an anti-syntaxin 18 antibody (green) (Fig. 10G to R). WT and ΔQ mutant PsVs had a similar colocalization with syntaxin 18 at approximately 40% (Fig. 10J, J1, N, N1, and S), indicated by the M1 coefficient. ΔIL mutant PsVs had a significantly reduced colocalization with syntaxin 18 at approximately 25% (Fig. 10R, R1, and S; *P* < 0.001). This data supports the idea that HPV16 PsVs will associate with syntaxin 18 during viral trafficking.

We next performed a co-immunoprecipitation of syntaxin 18 and the L1 capsid protein to explore the interaction between the WT and mutant PsVs and syntaxin 18. Precleared cell lysates were incubated with either an anti-syntaxin 18 antibody or rabbit IgG isotype overnight at 4°C before incubation with magnetic A/G beads. The Western blot shows that L1 was present in all sample flow throughs while syntaxin 18 was only found in the sample flow throughs that were incubated with the anti-syntaxin 18 antibody (Fig. 10T [Left]). After elution, the WT and ΔQ mutant PsV eluates both contained presence of the L1 protein and syntaxin 18, while the ΔIL mutant PsV eluate was lacking the L1 protein while still showing presence of syntaxin 18 (Fig. 10T [Right]). A nonspecific band was observed at the same size as the predicted heavy chain at 50 kDa in the CamVir-1 eluate blot, despite CamVir-1 being from a different species than the anti-syntaxin 18 antibody. These data suggest that the ΔIL mutant PsVs are unable to interact, either directly or indirectly, with syntaxin 18 after mutation of the isoleucine and leucine residues.

## DISCUSSION

This study supports that the minor capsid protein L2 is necessary for infection of HPV16. Mutation to the L2 sequence at residues 45 and 46 resulted in the inability of the virus to progress past the ER and enter the nucleus. These results confirm the importance of the 43-DQILQ-47 sequence as observed by Yan et al. (55). Yan et al. performed alanine scanning mutagenesis analyses on the 44-QIL-46 and 46-LQY-48 residues of CHO-K1 cells and observed a significant reduction in infection. Our alanine substitutions of the isoleucine and leucine residues is consistent with their results with the exception that, in our study, replacement of the leucine in residue 45 with alanine resulted in a reduction infection while Yan et al. replaced the leucine in residue 45 with lysine and did not observe a reduction in infection. A recent study published by the DiMaio lab also performed alanine-scanning mutagenesis of the 46L amino acid and found a significant reduction in infectivity (56).

Previous studies have determined the importance of BPV1 residues 40 to 44 DKILK during trafficking to the ER (35, 36). Antibody targeting of BPV1 L2 residues 40–44 resulted in noninfectious particles (35). This study observes the homologous sequence in HPV16. In HPV16, the BPV1 40-DKILK-44 sequence is conserved as 43-DQILQ-47. The DQ/KILQ/K sequence is conserved across human papillomaviruses, including both high- and low-risk types. Aspartic acid, isoleucine, and leucine are conserved with little to no variation, while either glutamine or lysine are found to flank the isoleucine-leucine residues. Based on our findings, substitution of glutamine at residue 44 results in infectious PsVs that follow a similar trafficking pattern to that of the WT L2 PsVs (Fig. 1). This does not suggest any particular involvement of glutamine at residue 44 in HPV16 trafficking.

HPV16 PsVs have been observed to associate with ER membrane proteins calnexin (37) and luminal ER proteins GRP78 and ERp29 (33, 37). These observations have been made as early as 4 hpi, increasing at 12 hpi (37), and as late as 20 hpi (33). Our results confirm ER and WT PsV association 20 hpi. Day et al. did not observe colocalization with calnexin 24 hpi (32), which may suggest that occurs earlier than 24 h.

Our findings show that substitution of the isoleucine and leucine residues at positions 45 and 46 with alanine results in a significant loss of infectivity of the HPV16 PsVs (Fig. 1C and E). The ΔIL and ΔQ mutant PsVs are similarly able to package the reporter plasmid and maintain similar L1 and L2 protein levels to the PsVs containing WT L2 (Fig. 2). There is also no difference between the WT, ΔIL mutant, and ΔQ mutant PsVs in regard to their ability to bind to the cell (Fig. 2E and F). WT, ΔIL mutant, and ΔQ mutant PsVs are all found to co-localize with early endosome marker EEA-1, suggesting that the lack of infectivity of the ΔIL mutant PsVs is not a result of the inability to internalize (Fig. 2E and G; Fig. 3).

Both ΔIL and ΔQ mutant PsVs follow a similar trafficking pattern to the WT L2 PsVs until 20 hpi. Our results further support that HPV16 PsVs will localize near the ER before establishing infection in the nucleus. WT, ΔIL, and ΔQ PsVs are found to overlap with the early endosome marker (EEA-1) at 4 hpi (Fig. 3), lysosome marker (LAMP-1) at 8 hpi (Fig. 5), Golgi apparatus marker (GM130) at 16 hpi (Fig. 6), ER transmembrane protein (calnexin) at 20 hpi (Fig. 7), and luminal ER protein marker (GRP78) at 20 hpi (Fig. 8). This suggests that, while L2 residues 45 and 46, as well as 44, are not directly or indirectly involved with the trafficking of HPV16 PsVs to reach the ER, L2 residues 45 and 46 are involved, either directly or indirectly, in mediating the trafficking of the viral particles past the ER toward the nucleus. It is currently unclear whether the ΔIL mutant PsVs are unable to traffic through the ER or are able to traffic to the ER but are unable to reach the nucleus during nuclear membrane breakdown. With immunofluorescence imaging and colocalization analysis, we were able to determine that ΔIL mutant PsVs follow a similar trafficking pattern to the WT PsVs up until the PsVs reach the ER. While WT PsVs are then able to interact with syntaxin 18, transport the pseudogenome to the nucleus, and unload the genome, ΔIL mutant PsVs are unable to interact with syntaxin 18, progress beyond the ER, and, as a result, accumulate outside of the nucleus. While we observed a complete reduction in infectivity for the ΔIL mutant PsVs, we did not observe a complete reduction in the colocalization between luminal protein GRP78 and the ΔIL mutant PsVs. Thus, while our data may suggest that the ΔIL mutant PsVs are unable to enter the ER, analysis using a higher resolution microscope is needed to further explore whether the decreased association between the ΔIL mutant PsVs and GRP78 is because of a lack of entry into the ER. We are actively working on observing the interaction with ER proteins at a higher microscope resolution.

After 48 hpi, the pseudogenome of the ΔIL mutant PsVs does not co-localize with the PML bodies as observed in the WT and ΔQ mutant PsVs. Instead, the ΔIL mutant PsV pseudogenome is found to co-localize with LAMP-1, suggesting a redirection of the ΔIL PsVs to the lysosome. After 96h, the remaining ΔIL PsVs are found to be degraded in the lysosome. This finding supports the loss of infectivity observed by flow cytometry analysis of the ΔIL mutant PsVs as the PsVs are unable to unload the pseudogenome into the nucleus.

Isoleucine- and leucine-based motifs (II, IL, and LL) have been found to be involved with vesicular sorting, particularly between both the ER and Golgi apparatus (47–50). The isoleucine-leucine motif is highly conserved across numerous papillomavirus types. It is possible that the isoleucine and leucine residues are important for localization of the HPV16 PsVs to the nucleus. More work is needed to determine the potential involvement of L2 residues 45 and 46 in nuclear localization. Similar residues to the DQILQ sequence can be found in exposed regions of other virus proteins, including bluetongue VP5, human immunodeficiency virus gp120, and picornavirus VP1 (57–60).

**FIG 11:**
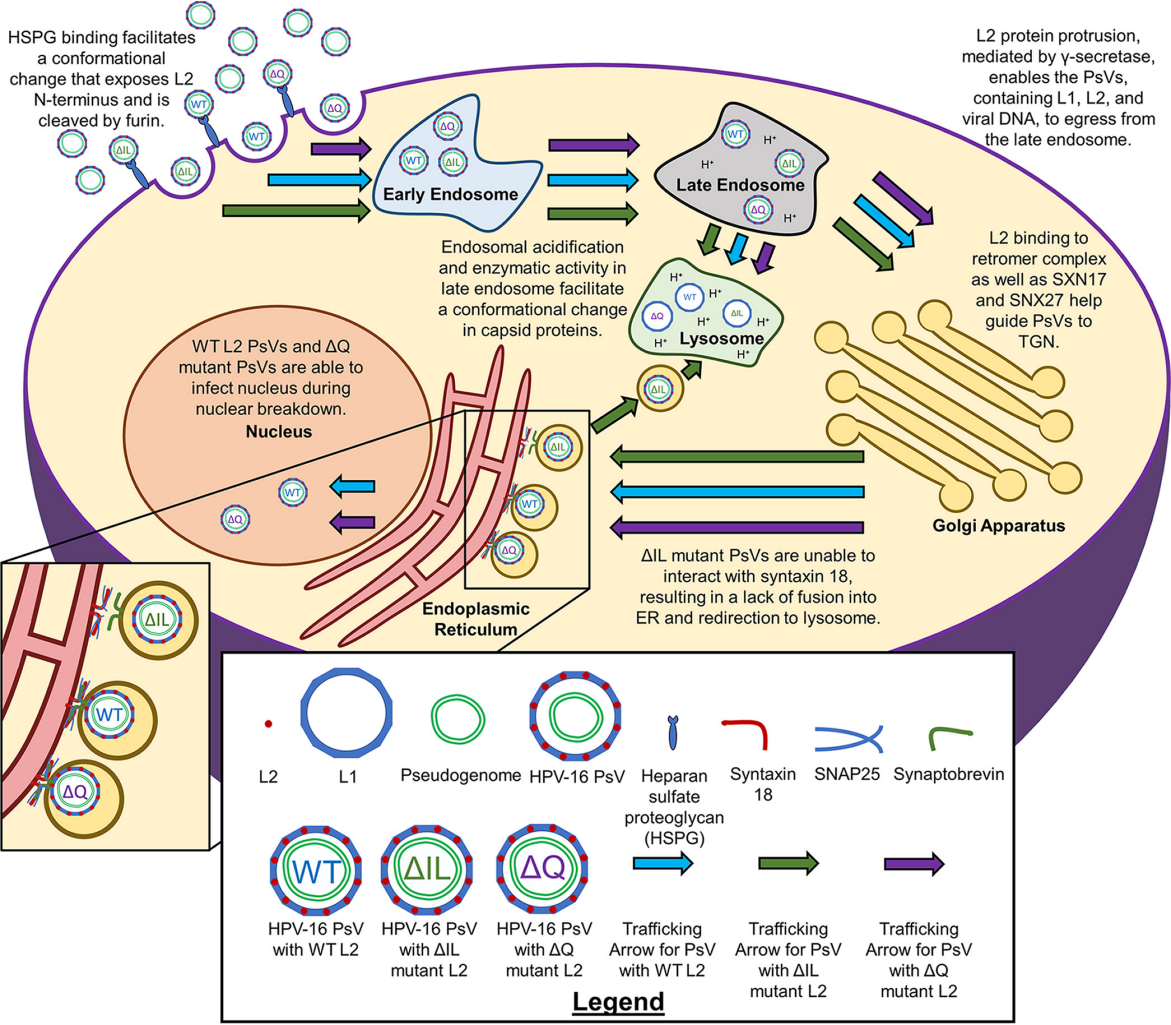
Cartoon model of proposed HPV16 trafficking. HPV16 PsVs enter the cell upon HSPG binding and furin cleavage of the exposed L2 N-terminus. PsVs travel to the early endosome, followed by the late endosome. The acidic pH of the late endosome and enzymes facilitate a conformational change in the capsid proteins. Some of the L1 protein is sent to the lysosome for degradation. The L2 protein facilitates the movement from the late endosome and retrograde trafficking to the trans-Golgi network, as well as the rest of the Golgi apparatus. L2 embeds itself into the endosome vesicle through mediation by γ-secretase, which facilitates the interaction with sorting proteins such as retromer, SNX17, and SNX27. PsVs approach the ER membrane and interact with syntaxin 18, allowing for vesicular fusion into the ER before nuclear breakdown during mitosis. The WT and ΔQ mutant PsVs infect the nucleus and deposit the pseudogenome. However, ΔIL mutant PsVs are unable to interact with syntaxin 18 and, thus, are unable to progress past the ER and are redirected to the lysosome.

A study involving BK polyomavirus (BKPyV) elucidated the involvement of syntaxin 18 in viral trafficking (60). BKPyV, a nonenveloped virus, is found to utilize endosomal protein rab18 to form a tethering complex with cytosolic proteins that attach to the ER membrane and bring the v-SNARE in proximity to syntaxin 18, allowing vesicle fusion to occur. Of course, the differences in viral trafficking observed in both BKPyV and HPV make it difficult to create direct parallels between them. Nonetheless, the reduction in HPV16 PsV infection observed when knocking down syntaxin 18 (Fig. 10) and the implication of syntaxin 18 in BKPyV trafficking raise an interesting question to explore. A potential mechanism for HPV16 viral trafficking is that the exposed isoleucine and leucine residues are involved in forming a tethering complex that bring the v-SNARE and syntaxin 18 together to allow for vesicular fusion and PsV entry into the ER. The mutated isoleucine and leucine residues would be unable to bind to an intermediate protein, be unable to form the tethering complex, be unable to enter the ER, and, thus, be unable to deposit the DNA into the nucleus (Fig. 11; model). Further studies need to be done to explore this possibility.

Recent studies support the retention of the major capsid L1 protein after movement from the lysosome instead of disassembly (10, 11, 30). The L1 protein has been found to associate with ER membrane protein calnexin and luminal chaperone protein ERp29 (37). Our data supports these previous data as we observed L1 colocalization with each organelle as well as consistent EdU and L1 colocalization throughout the trafficking pathway. It is still unclear the potential involvement of the L1 protein in the trafficking of HPV16 through the Golgi apparatus and ER. More work is needed to determine if the loss of infectivity in the ΔIL mutant PsVs can be directly attributed to the L2 protein or if it is the result of a loss of interaction between the L1 and L2 proteins.

In summary, we show that mutation at residues 45 and 46 in the 43-DQILQ-47 L2 protein reduces HPV16 PsV infection in HaCaT cells. Our results suggest that the 43-DQILQ-47 sequence, specifically the isoleucine and leucine residues, help traffic the viral DNA past the ER toward the nucleus. When the isoleucine and leucine residues are replaced, without any other changes in the L2 protein, the resulting PsVs are noninfectious. Substitution of the isoleucine and leucine residues with alanine results in the loss of infectivity and the redirecting of the PsVs to the lysosome. Additional studies of the 43-DQILQ-47 sequence in L2 will help further understand the role of the sequence in the intracellular trafficking of HPV in human keratinocytes.

## MATERIALS AND METHODS

### Cell culture.

HaCaT cells were purchased from AddexBio (San Diego, CA). HaCaT cells are an immortalized human keratinocyte cell line derived from adult human skin as described (61). Cells were cultured in Dulbecco’s modified Eagle’s medium (DMEM) (R&D Systems; Minneapolis, MN), supplemented with 10% fetal bovine serum (FBS) (GeminiBio; West Sacramento, CA).

### PsV production and pseudogenome packaging efficiency.

HPV16 PsVs were produced in 293TT cells as described by Buck et al. (17) and purified by ultracentrifugation at 303,000 × *g* for 210 min using a Beckman-Coulter L8-70M ultracentrifuge and a SW 55 Ti swinging-bucket rotor (Beckman Coulter; Brea, CA). Wild-type (WT) PsVs were produced using p16sheLL plasmid. ΔD, ΔQ, ΔI, ΔL, and ΔIL mutant PsVs were derived from p16SheLL with the substitution of specific L2 residues 43 (D), 44 (Q), 45 (I), 46 (L), or 45 and 46 (IL) using alanine scanning mutagenesis, performed with a Q5 site-directed mutagenesis kit (E0552S; New England BioLabs; Ipswich, MA) and 2720 Thermal Cycler (Applied Biosystems; Foster City, CA). Primers for the mutagenesis were obtained from Integrated DNA Technologies (IDT; Coralville, IA). GFP-encoding p8fwB plasmid was used as the reporter plasmid, hence referred to as the pseudogenome. p8fwB and p16sheLL plasmids were a generous gift from Dr. Schiller (NCI; Baltimore, MD). To generate PsVs labeled with 5-ethynyl-2’-deoxyridine (EdU), media was replaced and supplemented with 30 μM EdU at 12h posttransfection. Null (∅) plasmid preparation was prepared by subjecting 293TT cells to PsV production without the addition of p16sheLL or p8fwB plasmids. Identical Optiprep fractions were collected for ∅ plasmid preparation after ultracentrifugation and used as a negative control. As an additional negative control, a volume of 33% Optiprep equal to that of the WT PsVs was added to the cell culture.

Pseudogenome packaging efficiency was confirmed with quantitative PCR (qPCR; StepOne real-time PCR system; Applied Biosystems) for p8fwB plasmid after phenol-chloroform extraction of the pseudogenome. Purified viral fractions were added to a digestion mix containing 100 mM tris-HCl pH 7.5, 10 mM dithiothreitol (DTT; Thermo Fisher Scientific; Waltham, MA), 100 mM EDTA (Invitrogen; Carlsbad, CA), 1% SDS, and 0.2 mg Proteinase K (Invitrogen) and heated at 50°C for 15 min before addition of an equal volume of phenol:chloroform:isoamyl alcohol (25:24:1; MilliporeSigma; St. Louis, MO). Digested PsVs were centrifuged at 19,000 × *g* for 5 min. The upper aqueous layer was transferred to a new tube followed by addition of an equal volume of 3 M NaOAc pH 5. The resulting tube contents had five volumes of 100% ethanol added and were incubated overnight at −80°C. Tube contents were centrifuged at 14,000 × *g* for 30 min, the supernatant was discarded, and the pellet was resuspended in distilled water. Primers for p8fwB were obtained from Integrated DNA Technologies (IDT). All of the experiments in this study infected cells to achieve about 15% GFP expression, which corresponds to about 1500 viral genome equivalents (vge) per cell.

### Western blot and binding assay.

Primary antibodies were used at a dilution of 1:1000. Antibodies used were an anti-HPV L1 antibody (Cam Vir-1, MAB885; Chemicon International; Temecula, CA), an anti-HPV16 L2 antibody (2JG mab#5, sc-65709; Santa Cruz Biotechnologies; Dallas, TX), and an anti-actin antibody (A3853; MilliporeSigma) for normalization. IRDye secondary antibody 680 anti-mouse (926-68072; Li-Cor; Lincoln, NE) was used at a dilution of 1:30,000. HaCaT cells were seeded to the bottom of 6-well plates at a density of 200,000 cells per well for 24 h. After 24 h, cells were placed on ice for 30 min before addition of HPV16 PsVs for an additional 2 h. Cells were washed with 1x PBS to remove unbound virus and then incubated for an additional 2 h at 37°C. Cells were harvested using a lysis buffer containing Nonidet P-40 (United States Biological; Salem, MA) and 2% Protease Inhibitor Cocktail (Promega; Madison, WI). For the internalization assay, trypsin was added to the cells for 10 min to remove bound PsVs and the cells were then centrifuged at 600 × *g* for 5 min. Cells were washed three times with 1x PBS and then resuspended in lysis buffer. SDS-PAGE was performed using the lysates, and proteins were transferred to nitrocellulose membrane. Membranes were blocked at 4°C overnight with 5% milk in wash buffer containing tris-HCl pH 7.5 buffered saline with 0.1% Tween 20 (TBST; Thermo Fisher Scientific). Incubation with primary antibodies was performed at 4°C overnight. Incubation with secondary antibodies was performed at room temperature for 30 min. Protein bands were visualized using Li-Cor Odyssey Imaging System.

### Flow cytometry and infection assays.

HaCaT cells were seeded onto the bottom of 24-well plates at a density of 50,000 cells per well for 24 h. The experiment was performed in triplicate for each treatment. After 24 h, the cells were placed on ice for 30 min. HPV16 PsVs were allowed to bind on ice for 2 h, followed by washing with 1× PBS to remove unbound virus. Cells were incubated at 37°C for 48 h. Cells were harvested by addition of trypsin, followed by washing three times in 1× PBS with centrifugation at 600 × *g* for 5 min. Samples were analyzed for GFP expression with a flow cytometer (BD Accuri C6; BD Biosciences; Franklin Lakes, NJ). Ten thousand events were collected for each sample.

### Immunofluorescence microscopy.

HaCaT cells were seeded onto glass coverslips at a density of 50,000 cells per coverslip for 24 h. After 24 h, coverslips were placed on ice for 30 min. HPV16 PsVs were allowed to bind on ice for 2 h. Coverslips were washed three times with 1x PBS and were incubated at 37°C for 4, 8, 16, 20, or 48 h. Cells were fixed with 4% paraformaldehyde (PFA) at room temperature for 15 min. Cells were then permeabilized with a blocking buffer containing 3% BSA/PBS and 0.5% Triton X-100/PBS for 30 min, followed by extensive washes with 1× PBS. Coverslips were incubated with primary antibody diluted 1:100 in 3% BSA/PBS for 1 h. Coverslips were washed extensively with 1× PBS and then incubated with secondary antibody diluted at 1:2000 for 30 min. Coverslips were washed extensively with 1× PBS and the Click-It reaction was performed for 30 min using the Click-It Alexa Fluor 488 imaging kit (Thermo Fisher Scientific) as per the manufacturer’s instructions. Coverslips were mounted onto slides using Prolong Gold Antifade with 4’,6-diamidino-2-phenylindole (DAPI; Life Technologies; Carlsbad, CA).

Images were taken on a Leica TCS SP8 confocal microscope (Leica Microsystems; Wetzlar, Germany) using a 63× objective, a photomultiplier tube (PMT) sensor, and 405 Diode, 488 argon, DPSS 561, and HeNe 633 lasers. A set of Z-stacks were taken for each scan from the top of the cells to the bottom in 0.30-μm intervals. Sequential scans using four wavelength channels 408 nm, 488 nm, 561 nm, and 633 nm were used corresponding to the appropriate fluorophore attached to the secondary antibody; 408 nm wavelength was used to detect DAPI signal.

The following primary antibodies were used for immunofluorescence: anti-EEA-1 antibody (C-15, sc-6414; Santa Cruz Biotechnologies), anti-LAMP-1 (ab62562; abcam; Cambridge, United Kingdom), anti-GM130 (P-20, sc-16268; Santa Cruz Biotechnologies), anti-calnexin (PA534754; ThermoFisher Scientific), anti-GRP78 (ab21685; abcam), an anti-PML antibody (PG-M3; sc-966; Santa Cruz Biotechnologies), anti-HPV16 L1 antibodies (H16.V5 and 33L1-7) (a kind gift from Dr. Neil Christensen, Penn State University), and an anti-syntaxin 18 antibody (HPA003019; MilliporeSigma). The following secondary antibodies were used for immunofluorescence: donkey anti-rabbit 568, donkey anti-goat 568, and donkey anti-mouse 647. All secondary antibodies were purchased from Life Technologies.

### siRNA Transfection.

Single interfering RNA (siRNA) targeting syntaxin 18 was purchased from Santa Cruz Biotechnologies (sc-63092). Experiments including siRNA used a universal scrambled siRNA duplex as a negative control (SR300004; Origene Technologies Inc., Rockville, MD, USA). HaCaT cells were plated into a 24-well plate at a density of 40,000 cells per well for 24 h, and 30 pmol of either syntaxin 18 or scrambled siRNA was added to each well using Lipofectamine RNAiMAX reagent as per the manufacturer’s instructions (Thermo Fisher Scientific). Each treatment was performed in triplicate. Twelve-hour posttransfection, either syntaxin 18 or scrambled siRNA was added to each well again under the same conditions. Cells were collected at 24, 48, and 72 h after the initial transfection and were subjected to 10% SDS-PAGE and Western blot analysis for syntaxin 18 and actin proteins.

For the infectivity assay involving siRNA, cells were placed on ice for 30 min 24 h after initial transfection. HPV16 WT PsVs were allowed to bind on ice for 2 h and were then washed with 1× PBS to remove unbound virus. The cells were placed in a 37°C incubator for 48 h before processing for flow cytometry. The experiment was performed in quadruplicate for each treatment.

### Immunoprecipitation.

HaCaT cells were seeded to the bottom of a T150 flask at a density of 4.5 × 10^6^ cells per flask and were incubated for 24 h at 37°C. After 24 h, cells were placed on ice for 30 min. HPV16 WT or mutant PsVs were allowed to bind on ice for 2 h, followed by washing with 1x PBS to remove unbound virus. The cells were placed in a 37°C incubator for 20 h before harvesting with trypsin. Harvested cells were centrifuged at 600 × *g* for 5 min and washed three times with 1× PBS. Cells were lysed using a lysis buffer containing Nonidet P-40 (United States Biological) and 2% Protease Inhibitor Cocktail (Promega) and were then placed on ice for 1 h. Lysed cells were passed through a 22-gauge needle 10 times before centrifugation at 10,000 × *g* for 10 min. The supernatant was precleared with Pierce Protein A/G Magnetic Beads (ThermoFisher Scientific) overnight at 4°C with a tube rotator. The supernatant was separated from the magnetic A/G beads using a magnetic tube rack, and 3.5 μg of either rabbit IgG isotype or anti-syntaxin 18 antibody (PA5-112291; Invitrogen) was incubated with the supernatant overnight at 4°C with a tube rotator. Magnetic A/G beads were added to the antibody-supernatant mixture and were incubated at 4°C for 4 h using a tube rotator. Magnetic beads were pelleted using a magnetic tube rack and the supernatant was separated and labeled as “Flow Through”. The pellet was washed five times with lysis buffer before addition of Laemmli sample buffer containing 125 mM tris-HCl pH 6.8, 4% SDS, 20% glycerol (MilliporeSigma), and 10% β-mercaptoethanol (MilliporeSigma). Samples were boiled for 7 min at 95°C and then placed onto a magnetic tube rack. The supernatant was removed from the bead pellet and labeled as “Eluate”. Flow Through and Eluate samples were subjected to 10% SDS-PAGE and Western blot analysis for presence of HPV16 L1 protein and syntaxin 18 protein. Primary antibodies used were: anti-HPV L1 antibody (Cam Vir-1, MAB885; Chemicon International) and anti-syntaxin 18 antibody (PA5-112291; Invitrogen).

### Colocalization analysis.

Analysis of confocal images was performed using the JACoP plugin for ImageJ (62) as described (63). Colocalization was determined using the Manders’ coefficient M1, being the percentage of overlap of EdU-labeled pseudogenome or HPV16 L1 protein with a time-specific organelle. Thresholds were set manually and kept constant throughout all analyses. Four slices from the center of the Z-stack were selected for each analysis. M1 coefficients with error bars represented by the SEM were determined using the average of the four Z-stack slices for six independent scans.
